# Supplementary material for: A systematic review and meta-analysis of the impact of vaccination on prevention of long COVID
Source: Nat Commun. 2025 Nov 24;16:10326. doi: 10.1038/s41467-025-65302-0 (PMC12644529; doi:10.1038/s41467-025-65302-0)
Supplement: Supplementary file 1 — Supplementary Information [file 41467_2025_65302_MOESM1_ESM.docx]

# Supplementary materials

1. **Supplementary Methods**
   1. **Search strategy**

**Databases searched:**

- Embase (Ovid): 2022-2024/03/01
- Medline ALL (Ovid): 2022-2024/03/01
- PubMed (NLM): 2022-2024/03/01
- LILACS (www): 2022-2024/03/01
- Europe PMC, includes BioRxiv & medRxiv (www): 2022-2024/03/01
- WHO Covid-19 database^a^ (www): 2022-2024/01/25
- Cochrane COVID Register^b^ (www): 2022-2024/02/02

^a^WHO COVID-19 database ceased on June 2023, therefore update searches were not necessary; ^b^Cochrane COVID-9 Study Register ceased at the end of January 2024, therefore update searches were not necessary

**Embase (Ovid): 2022-2024/01/08**

**Searched 9.1.24**

1 immunization/ or active immunization/ or mass immunization/ or vaccination/ or vaccination coverage/ or vaccine failure/ 324831

2 revaccination/ 3068

3 (immunis$ or immuniz$ or immunity or vaccin$ or jab or jabs or shot or shots or booster or boosters or revaccin$ or unvaccin$).ti,ab,ot,kf,kw. 885844

4 or/1-3 940180

5 (PASC or "chronic covid syndrome$").ti,ab,ot,kf,kw. 1139

6 ("post acute" adj2 sequela$ adj2 (covid or coronavirus or coronovirus or "corona virus" or COV)).ti,ab,ot,kw. 476

7 (("long$ term$" or longterm$ or "long$ haul$" or longhaul$ or "long$ tail$" or longtail$ or longduration$ or "long duration$" or longlast$ or "long last$" or longstanding$ or "long standing$" or "medium$ term$" or mediumterm$ or "late effect$" or recurren$ or prolong$ or post-viral$ or chronic$ or postacute or "post acute" or persistent$) adj3 (covid$ or coronavirus$ or corona$ virus$ or coronovirus$ or corono$ virus$ or coronavirinae$ or corona$ virinae$ or Cov or "2019-nCoV$" or 2019nCoV$ or "19-nCoV$" or 19nCoV$ or nCoV2019$ or "nCoV-2019$" or nCoV19$ or "nCoV-19$" or "HCoV-19$" or HCoV19$ or "HCoV-2019$" or HCoV2019$ or "2019 novel$" or Ncov$ or "n-cov" or "SARS-CoV-2$" or "SARSCoV-2$" or "SARSCoV2$" or "SARS-CoV2$" or SARSCov19$ or "SARS-Cov19$" or "SARSCov-19$" or "SARS-Cov-19$" or SARSCov2019$ or "SARS-Cov2019$" or "SARSCov-2019$" or "SARS-Cov-2019$" or SARS2$ or "SARS-2$" or SARScoronavirus2$ or "SARS-coronavirus-2$" or "SARScoronavirus 2$" or "SARS coronavirus2$" or SARScoronovirus2$ or "SARS-coronovirus-2$" or "SARScoronovirus 2$" or "SARS coronovirus2$" or "severe acute respiratory syndrome$")).ti,ab,ot,kw. 10596

8 (longcovid$ or long covid$ or longcoronavirus$ or longcorona$ virus$ or long coronavirus$ or long corona$ virus$ or longcoronovirus$ or longcorono$ virus$ or long coronovirus$ or long corono$ virus$ or longcoronavirinae$ or longcorona$ virinae$ or long coronavirinae$ or long corona$ virinae$ or longCov or long Cov or longsars$ or long sars$ or "long severe acute respiratory syndrome$" or longncov$ or long ncov$ or longhcov$ or long hcov$ or "post-acute covid" or "post-acute corona$" or "post-acute corono$" or "post-acute cov" or "post-acute ncov" or "post-acute sars" or "post-acute severe respiratory syndrome$").ti,ab,ot,kw. 6458

9 ((long$ or endur$ or legacy$ or slow$ or gradual$ or protract$ or lengthy$ or chronic$ or persist$ or relaps$ or remit$ or remission$ or residual$ or delay$ or prolong$ or extend$ or linger$ or permanent$ or fluctuat$ or sequela$ or multisystem$ or "multi system$" or nonrecover$ or "non recover$" or subacute$ or "sub acute$" or lasting$ or continuous$ or continual$ or continuing$ or postacute$ or "post acute$" or postdischarg$ or "post discharg$" or postinfect$ or "post infect$" or postviral$ or "post viral$" or postvirus$ or "post virus$" or "late effect$") adj2 (covid$ or coronavirus$ or corona$ virus$ or coronovirus$ or corono$ virus$ or coronavirinae$ or corona$ virinae$ or Cov or "2019-nCoV$" or 2019nCoV$ or "19-nCoV$" or 19nCoV$ or nCoV2019$ or "nCoV-2019$" or nCoV19$ or "nCoV-19$" or "HCoV-19$" or HCoV19$ or "HCoV-2019$" or HCoV2019$ or "2019 novel$" or Ncov$ or "n-cov" or "SARS-CoV-2$" or "SARSCoV-2$" or "SARSCoV2$" or "SARS-CoV2$" or SARSCov19$ or "SARS-Cov19$" or "SARSCov-19$" or "SARS-Cov-19$" or SARSCov2019$ or "SARS-Cov2019$" or "SARSCov-2019$" or "SARS-Cov-2019$" or SARS2$ or "SARS-2$" or SARScoronavirus2$ or "SARS-coronavirus-2$" or "SARScoronavirus 2$" or "SARS coronavirus2$" or SARScoronovirus2$ or "SARS-coronovirus-2$" or "SARScoronovirus 2$" or "SARS coronovirus2$" or "severe acute respiratory syndrome$")).ti,ab,ot,kw. 17052

10 (("long$ term$" or longterm$ or "long$ haul$" or longhaul$ or "long$ tail$" or longtail$ or longduration$ or "long duration$" or longlast$ or "long last$" or longstanding$ or "long standing$" or "medium$ term$" or mediumterm$ or "post-virus$" or "post-viral") adj3 (covid$ or coronavirus$ or corona$ virus$ or coronovirus$ or corono$ virus$ or coronavirinae$ or corona$ virinae$ or Cov or "2019-nCoV$" or 2019nCoV$ or "19-nCoV$" or 19nCoV$ or nCoV2019$ or "nCoV-2019$" or nCoV19$ or "nCoV-19$" or "HCoV-19$" or HCoV19$ or "HCoV-2019$" or HCoV2019$ or "2019 novel$" or Ncov$ or "n-cov" or "SARS-CoV-2$" or "SARSCoV-2$" or "SARSCoV2$" or "SARS-CoV2$" or SARSCov19$ or "SARS-Cov19$" or "SARSCov-19$" or "SARS-Cov-19$" or SARSCov2019$ or "SARS-Cov2019$" or "SARSCov-2019$" or "SARS-Cov-2019$" or SARS2$ or "SARS-2$" or SARScoronavirus2$ or "SARS-coronavirus-2$" or "SARScoronavirus 2$" or "SARS coronavirus2$" or SARScoronovirus2$ or "SARS-coronovirus-2$" or "SARScoronovirus 2$" or "SARS coronovirus2$" or "severe acute respiratory syndrome$")).ti,ab,ot,kw. 3714

11 ((postcovid$ or post covid$ or postcoronavirus$ or postcorona$ virus$ or post coronavirus$ or post corona$ virus$ or postcoronovirus$ or postcorono$ virus$ or post coronovirus$ or post corono$ virus$ or postcoronavirinae$ or postcorona$ virinae$ or post coronavirinae$ or post corona$ virinae$ or postCov or post Cov or postsars$ or post sars$ or "post severe acute respiratory syndrome$" or postncov$ or post ncov$ or posthcov$ or post hcov$) adj3 (syndrome$ or disorder$ or illness$ or sickness$ or disease$ or condition$ or symptom$ or sign$ or prognos$ or followup$ or "follow up$" or feature$ or comorbid$ or "co morbid$" or multimorbid$ or "multi morbid$" or survivor$ or survival$ or risk$ or care$ or convalescen$ or recuperat$ or aftercare$ or ambulatory$ or outpatient$ or "out patient$")).ti,ab,ot,kw. 4125

12 ((ongoing$ or long$ or endur$ or legacy$ or slow$ or gradual$ or protract$ or lengthy$ or chronic$ or persist$ or relaps$ or remit$ or remission$ or residual$ or delay$ or prolong$ or extend$ or linger$ or permanent$ or fluctuat$ or multisystem$ or "multi system$" or nonrecover$ or "non recover$" or subacute$ or "sub acute$" or lasting$ or continuous$ or continual$ or continuing$ or postacute$ or "post acute$" or postdischarg$ or "post discharg$" or postinfect$ or "post infect$" or postviral$ or "post viral$" or postvirus$ or "post virus$" or "medium$ term$" or mediumterm$) adj4 (sequela$ or illness$ or symptom$ or sign$ or prognos$ or rehab$ or convalescen$ or recuperat$ or followup$ or "follow up$" or feature$) adj10 (covid$ or coronavirus$ or corona$ virus$ or coronovirus$ or corono$ virus$ or coronavirinae$ or corona$ virinae$ or Cov or "2019-nCoV$" or 2019nCoV$ or "19- nCoV$" or 19nCoV$ or nCoV2019$ or "nCoV-2019$" or nCoV19$ or "nCoV-19$" or "HCoV-19$" or HCoV19$ or "HCoV-2019$" or HCoV2019$ or "2019 novel$" or Ncov$ or "n-cov" or "SARS-CoV-2$" or "SARSCoV-2$" or "SARSCoV2$" or "SARS-CoV2$" or SARSCov19$ or "SARS-Cov19$" or "SARSCov-19$" or "SARS-Cov-19$" or SARSCov2019$ or "SARS-Cov2019$" or "SARSCov-2019$" or "SARS-Cov-2019$" or SARS2$ or "SARS-2$" or SARScoronavirus2$ or "SARS-coronavirus-2$" or "SARScoronavirus 2$" or "SARS coronavirus2$" or SARScoronovirus2$ or "SARS-coronovirus-2$" or "SARScoronovirus 2$" or "SARS coronovirus2$" or "severe acute respiratory syndrome$")).ti,ab,ot,kw. 8927

13 or/5-12 26634

14 ((chronic$ or Long or post-acute or longterm or late or persistent$) adj5 (sequela$ or effect$ or symptom$)).ti,ab,ot,kf,kw. 410115

15 sars-related coronavirus/ or exp Severe acute respiratory syndrome coronavirus 2/ 107363

16 exp coronavirus disease 2019/ 376660

17 (coronavirinae/ or betacoronavirus/ or coronavirus infection/) and (epidemic/ or pandemic/) 10708

18 (Coronavirus$ or "covid 19" or 2019-ncov).ti,ab,kw,kf,ot. 432354

19 (2019-ncov or 2019ncov or corona-virus$ or cov19 or cov-19 or 19nCoV or COVID19 or COVID2019 or "Covid 2019").ti,ab,kw,kf,ot. 15890

20 (ncov$ or "sars cov$" or sarscov$ or "sars coronavirus$" or coronovirus$ or corono$ virus$ or "19-nCoV$" or 19nCoV$).ti,ab,kw,kf,ot. 161417

21 (SARS2$ or "SARS-2$" or SARScoronavirus$ or SARS-coronavirus$ or SARScoronovirus$ or SARS-coronovirus$).ti,ab,kw,kf,ot. 2922

22 ("HCoV-19$" or HCoV19$ or "HCoV-2019$" or HCoV2019$).ti,ab,kw,kf,ot. 70

23 ("2019 novel$" or Ncov$).ti,ab,kw,kf,ot. 5863

24 ("Severe Acute Respiratory Syndrome Coronavirus 2" or "Severe Acute Respiratory Syndrome Corona Virus 2").ti,ab,kw,kf,ot. 36929

25 exp "SARS-CoV-2 (lineage B.1.1)"/ 8289

26 exp "SARS-CoV-2 Omicron"/ 7345

27 (omikron or Omicron or "B.1.1.529" or "B11529" or xbb$).af. 13714

28 or/15-27 500310

29 14 and 28 10029

30 13 or 29 30508

31 4 and 30 5751

32 animal/ or animal experiment/ 4726380

33 (rat or rats or mouse or mice or murine or rodent or rodents or hamster or hamsters or pig or pigs or porcine or rabbit or rabbits or animal or animals or dogs or dog or cats or cow or bovine or sheep or ovine or monkey or monkeys).ti,ab,ot,hw. 7733732

34 32 or 33 7733732

35 human experiment/ or exp humans/ 26021849

36 34 not (34 and 35) 5786701

37 31 not 36 5666

38 limit 37 to yr="2022 -Current" 4444

**39 38 not (letter or editorial or conference or "conference abstract" or "conference paper" or "conference review").pt. 3497**

***The Embase strategy was updated on 1.2.24 (204 records) and 1.3.24 (226 records).***

*COVID facet based on terms from:*

World Health Organization (26 May 2021) WHO COVID-19 Database Search Strategy. Systematic search of the COVID-19 literature performed Monday through Friday for the WHO Database. Search strategy as of 26 May 2021. Searches performed by Tomas Allen, Kavita Kothari, and Martha Knuth. Available from: <https://www.who.int/docs/default-source/coronaviruse/who-covid-19-database/who-covid-19_sources_searchstrategy_20210526.pdf?sfvrsn=65209cc2_5>

Canadian Agency for Drugs and Technologies in Health (2.9.21) CADTH COVID-19 Search Strings: COVID-19 — EMBASE (Internet). Available from: <https://covid.cadth.ca/literature-searching-tools/cadth-covid-19-search-strings/>

NICE (18 December 2020) [accessed 17.8.21] COVID-19 rapid guideline: managing the long-term effects of COVID-19 [NG188]. Search history record [PDF]. NICE: London. Available from: <https://www.nice.org.uk/guidance/ng188/evidence/search-strategies-pdf-8957634445>

**Medline ALL (Ovid): 2022-2024/01/23**

**Searched 24.1.24**

1 exp Immunization/ 213463

2 (immunis$ or immuniz$ or immunity or vaccin$ or jab or jabs or shot or shots or booster or boosters or revaccin$ or unvaccin$).ti,ab,ot,kf,kw. 747942

3 or/1-2 805424

4 Post-Acute COVID-19 Syndrome/ 2922

5 (PASC or "chronic covid syndrome$").ti,ab,ot,kf,kw. 867

6 ("post acute" adj2 sequela$ adj2 (covid or coronavirus or coronovirus or "corona virus" or COV)).ti,ab,ot,kw. 342

7 (("long$ term$" or longterm$ or "long$ haul$" or longhaul$ or "long$ tail$" or longtail$ or longduration$ or "long duration$" or longlast$ or "long last$" or longstanding$ or "long standing$" or "medium$ term$" or mediumterm$ or "late effect$" or recurren$ or prolong$ or post-viral$ or chronic$ or postacute or "post acute" or persistent$) adj3 (covid$ or coronavirus$ or corona$ virus$ or coronovirus$ or corono$ virus$ or coronavirinae$ or corona$ virinae$ or Cov or "2019-nCoV$" or 2019nCoV$ or "19-nCoV$" or 19nCoV$ or nCoV2019$ or "nCoV-2019$" or nCoV19$ or "nCoV-19$" or "HCoV-19$" or HCoV19$ or "HCoV-2019$" or HCoV2019$ or "2019 novel$" or Ncov$ or "n-cov" or "SARS-CoV-2$" or "SARSCoV-2$" or "SARSCoV2$" or "SARS-CoV2$" or SARSCov19$ or "SARS-Cov19$" or "SARSCov-19$" or "SARS-Cov-19$" or SARSCov2019$ or "SARS-Cov2019$" or "SARSCov-2019$" or "SARS-Cov-2019$" or SARS2$ or "SARS-2$" or SARScoronavirus2$ or "SARS-coronavirus-2$" or "SARScoronavirus 2$" or "SARS coronavirus2$" or SARScoronovirus2$ or "SARS-coronovirus-2$" or "SARScoronovirus 2$" or "SARS coronovirus2$" or "severe acute respiratory syndrome$")).ti,ab,ot,kw. 9052

8 (longcovid$ or long covid$ or longcoronavirus$ or longcorona$ virus$ or long coronavirus$ or long corona$ virus$ or longcoronovirus$ or longcorono$ virus$ or long coronovirus$ or long corono$ virus$ or longcoronavirinae$ or longcorona$ virinae$ or long coronavirinae$ or long corona$ virinae$ or longCov or long Cov or longsars$ or long sars$ or "long severe acute respiratory syndrome$" or longncov$ or long ncov$ or longhcov$ or long hcov$ or "post-acute covid" or "post-acute corona$" or "post-acute corono$" or "post-acute cov" or "post-acute ncov" or "post-acute sars" or "post-acute severe respiratory syndrome$").ti,ab,ot,kw. 5205

9 ((long$ or endur$ or legacy$ or slow$ or gradual$ or protract$ or lengthy$ or chronic$ or persist$ or relaps$ or remit$ or remission$ or residual$ or delay$ or prolong$ or extend$ or linger$ or permanent$ or fluctuat$ or sequela$ or multisystem$ or "multi system$" or nonrecover$ or "non recover$" or subacute$ or "sub acute$" or lasting$ or continuous$ or continual$ or continuing$ or postacute$ or "post acute$" or postdischarg$ or "post discharg$" or postinfect$ or "post infect$" or postviral$ or "post viral$" or postvirus$ or "post virus$" or "late effect$") adj2 (covid$ or coronavirus$ or corona$ virus$ or coronovirus$ or corono$ virus$ or coronavirinae$ or corona$ virinae$ or Cov or "2019-nCoV$" or 2019nCoV$ or "19-nCoV$" or 19nCoV$ or nCoV2019$ or "nCoV-2019$" or nCoV19$ or "nCoV-19$" or "HCoV-19$" or HCoV19$ or "HCoV-2019$" or HCoV2019$ or "2019 novel$" or Ncov$ or "n-cov" or "SARS-CoV-2$" or "SARSCoV-2$" or "SARSCoV2$" or "SARS-CoV2$" or SARSCov19$ or "SARS-Cov19$" or "SARSCov-19$" or "SARS-Cov-19$" or SARSCov2019$ or "SARS-Cov2019$" or "SARSCov-2019$" or "SARS-Cov-2019$" or SARS2$ or "SARS-2$" or SARScoronavirus2$ or "SARS-coronavirus-2$" or "SARScoronavirus 2$" or "SARS coronavirus2$" or SARScoronovirus2$ or "SARS-coronovirus-2$" or "SARScoronovirus 2$" or "SARS coronovirus2$" or "severe acute respiratory syndrome$")).ti,ab,ot,kw. 14885

10 (("long$ term$" or longterm$ or "long$ haul$" or longhaul$ or "long$ tail$" or longtail$ or longduration$ or "long duration$" or longlast$ or "long last$" or longstanding$ or "long standing$" or "medium$ term$" or mediumterm$ or "post-virus$" or "post-viral") adj3 (covid$ or coronavirus$ or corona$ virus$ or coronovirus$ or corono$ virus$ or coronavirinae$ or corona$ virinae$ or Cov or "2019-nCoV$" or 2019nCoV$ or "19-nCoV$" or 19nCoV$ or nCoV2019$ or "nCoV-2019$" or nCoV19$ or "nCoV-19$" or "HCoV-19$" or HCoV19$ or "HCoV-2019$" or HCoV2019$ or "2019 novel$" or Ncov$ or "n-cov" or "SARS-CoV-2$" or "SARSCoV-2$" or "SARSCoV2$" or "SARS-CoV2$" or SARSCov19$ or "SARS-Cov19$" or "SARSCov-19$" or "SARS-Cov-19$" or SARSCov2019$ or "SARS-Cov2019$" or "SARSCov-2019$" or "SARS-Cov-2019$" or SARS2$ or "SARS-2$" or SARScoronavirus2$ or "SARS-coronavirus-2$" or "SARScoronavirus 2$" or "SARS coronavirus2$" or SARScoronovirus2$ or "SARS-coronovirus-2$" or "SARScoronovirus 2$" or "SARS coronovirus2$" or "severe acute respiratory syndrome$")).ti,ab,ot,kw. 3219

11 ((postcovid$ or post covid$ or postcoronavirus$ or postcorona$ virus$ or post coronavirus$ or post corona$ virus$ or postcoronovirus$ or postcorono$ virus$ or post coronovirus$ or post corono$ virus$ or postcoronavirinae$ or postcorona$ virinae$ or post coronavirinae$ or post corona$ virinae$ or postCov or post Cov or postsars$ or post sars$ or "post severe acute respiratory syndrome$" or postncov$ or post ncov$ or posthcov$ or post hcov$) adj3 (syndrome$ or disorder$ or illness$ or sickness$ or disease$ or condition$ or symptom$ or sign$ or prognos$ or followup$ or "follow up$" or feature$ or comorbid$ or "co morbid$" or multimorbid$ or "multi morbid$" or survivor$ or survival$ or risk$ or care$ or convalescen$ or recuperat$ or aftercare$ or ambulatory$ or outpatient$ or "out patient$")).ti,ab,ot,kw. 3028

12 ((ongoing$ or long$ or endur$ or legacy$ or slow$ or gradual$ or protract$ or lengthy$ or chronic$ or persist$ or relaps$ or remit$ or remission$ or residual$ or delay$ or prolong$ or extend$ or linger$ or permanent$ or fluctuat$ or multisystem$ or "multi system$" or nonrecover$ or "non recover$" or subacute$ or "sub acute$" or lasting$ or continuous$ or continual$ or continuing$ or postacute$ or "post acute$" or postdischarg$ or "post discharg$" or postinfect$ or "post infect$" or postviral$ or "post viral$" or postvirus$ or "post virus$" or "medium$ term$" or mediumterm$) adj4 (sequela$ or illness$ or symptom$ or sign$ or prognos$ or rehab$ or convalescen$ or recuperat$ or followup$ or "follow up$" or feature$) adj10 (covid$ or coronavirus$ or corona$ virus$ or coronovirus$ or corono$ virus$ or coronavirinae$ or corona$ virinae$ or Cov or "2019-nCoV$" or 2019nCoV$ or "19- nCoV$" or 19nCoV$ or nCoV2019$ or "nCoV-2019$" or nCoV19$ or "nCoV-19$" or "HCoV-19$" or HCoV19$ or "HCoV-2019$" or HCoV2019$ or "2019 novel$" or Ncov$ or "n-cov" or "SARS-CoV-2$" or "SARSCoV-2$" or "SARSCoV2$" or "SARS-CoV2$" or SARSCov19$ or "SARS-Cov19$" or "SARSCov-19$" or "SARS-Cov-19$" or SARSCov2019$ or "SARS-Cov2019$" or "SARSCov-2019$" or "SARS-Cov-2019$" or SARS2$ or "SARS-2$" or SARScoronavirus2$ or "SARS-coronavirus-2$" or "SARScoronavirus 2$" or "SARS coronavirus2$" or SARScoronovirus2$ or "SARS-coronovirus-2$" or "SARScoronovirus 2$" or "SARS coronovirus2$" or "severe acute respiratory syndrome$")).ti,ab,ot,kw. 6706

13 or/4-12 21997

14 ((chronic$ or Long or post-acute or longterm or late or persistent$) adj5 (sequela$ or effect$ or symptom$)).ti,ab,ot,kf,kw. 300919

15 exp Severe acute respiratory syndrome-related coronavirus/ 167482

16 COVID-19/ 253025

17 Coronavirus Infections/ 46122

18 (coronaviridae/ or exp coronavirus/ or betacoronavirus/ or exp betacoronavirus 1/) and (epidemics/ or pandemics/ or Disease Outbreaks/) 76944

19 (Coronavirus$ or "covid 19" or 2019-ncov).ti,ab,kw,kf,ot. 388465

20 (2019-ncov or 2019ncov or corona-virus$ or cov19 or cov-19 or 19nCoV or COVID19 or COVID2019 or "Covid 2019").ti,ab,kw,kf,ot. 10230

21 (ncov$ or "sars cov$" or sarscov$ or "sars coronavirus$" or coronovirus$ or corono$ virus$ or "19-nCoV$" or 19nCoV$).ti,ab,kw,kf,ot. 141131

22 (SARS2$ or "SARS-2$" or SARScoronavirus$ or SARS-coronavirus$ or SARScoronovirus$ or SARS-coronovirus$).ti,ab,kw,kf,ot. 2636

23 ("HCoV-19$" or HCoV19$ or "HCoV-2019$" or HCoV2019$).ti,ab,kw,kf,ot. 66

24 ("2019 novel$" or Ncov$).ti,ab,kw,kf,ot. 5245

25 ("Severe Acute Respiratory Syndrome Coronavirus 2" or "Severe Acute Respiratory Syndrome Corona Virus 2").ti,ab,kw,kf,ot. 37116

26 (omikron or Omicron or "B.1.1.529" or "B11529" or xbb$).af. 10018

27 or/15-26 422651

28 14 and 27 7756

29 13 or 28 24967

30 3 and 29 4424

31 animals/ not (animals/ and humans/) 5155785

32 30 not 31 4394

33 32 not (case reports or clinical conference or comment or editorial or letter).pt. 3846

**34 limit 33 to yr="2022 -Current" 2944**

***The Medline ALL strategy was updated on 1.2.24 (102 records) and 1.3.24 (219 records).***

*COVID facet based on terms from:*

World Health Organization (26 May 2021) WHO COVID-19 Database Search Strategy. Systematic search of the COVID-19 literature performed Monday through Friday for the WHO Database. Search strategy as of 26 May 2021. Searches performed by Tomas Allen, Kavita Kothari, and Martha Knuth. Available from: <https://www.who.int/docs/default-source/coronaviruse/who-covid-19-database/who-covid-19_sources_searchstrategy_20210526.pdf?sfvrsn=65209cc2_5>

Canadian Agency for Drugs and Technologies in Health (2.9.21) CADTH COVID-19 Search Strings: COVID-19 — EMBASE (Internet). Available from: <https://covid.cadth.ca/literature-searching-tools/cadth-covid-19-search-strings/>

NICE (18 December 2020) [accessed 17.8.21] COVID-19 rapid guideline: managing the long-term effects of COVID-19 [NG188]. Search history record [PDF]. NICE: London. Available from: <https://www.nice.org.uk/guidance/ng188/evidence/search-strategies-pdf-8957634445>

**PubMed (NLM): 2022-2024/01/24**

**Searched 24.1.24**

[**https://pubmed.ncbi.nlm.nih.gov/**](https://pubmed.ncbi.nlm.nih.gov/)

**24 #22 AND #23 774**

23 ("2022/01/01"[Date - Publication] : "3000"[Date - Publication]) 3,346,180

22 #20 NOT #21 1,101

21 LETTER[Publication Type] OR EDITORIAL[Publication Type] OR COMMENT[Publication Type] 2,218,993

20 #18 AND #19 1,111

19 pubstatusaheadofprint OR publisher[sb] OR pubmednotmedline[sb] 5,657,369

18 #17 NOT #16 3,711

17 #10 AND #13 3,877

16 #14 NOT (#14 AND #15) 3,770,128

15 Human[tiab] OR humans[tiab] 3,265,545

14 rat[tiab] OR rats[tiab] OR mouse[tiab] OR mice[tiab] OR murine[tiab] OR rodent[tiab] OR rodents[tiab] OR hamster[tiab] OR hamsters[tiab] OR pig[tiab] OR pigs[tiab] OR porcine[tiab] OR rabbit[tiab] OR rabbits[tiab] OR animal[tiab] OR animals[tiab] OR dogs[tiab] OR dog[tiab] OR cats[tiab] OR cow[tiab] OR bovine[tiab] OR sheep[tiab] OR ovine[tiab] OR monkey[tiab] OR monkeys[tiab] 4,725,135

13 #11 OR #12 910,777

12 (immunised[Text Word] OR immunise[Text Word] OR immunisation[Text Word] OR immunisations[Text Word] OR immunized[Text Word] OR immunize[Text Word] OR immunization[Text Word] OR immunizations[Text Word] OR immunity[Text Word] OR vaccine[Text Word] OR vaccines[Text Word] OR vaccination[Text Word] OR vaccinations[Text Word] OR vaccinated[Text Word] OR jab[Text Word] OR jabs[Text Word] OR shot[Text Word] OR shots[Text Word] OR booster[Text Word] OR boosters[Text Word] OR revaccination[Text Word] OR revaccinations[Text Word] OR revaccinated[Text Word] OR unvaccinated[Text Word]) 893,875

11 "Immunization"[Mesh] 213,271

10 #1 OR #2 OR #3 OR #4 OR #5 OR #6 OR #7 OR #8 OR #9 16,571

9 ((ongoing[Text Word] OR long[Text Word] OR endur[Text Word] OR legacy[Text Word] OR slow[Text Word] OR gradual[Text Word] OR protracted[Text Word] OR lengthy[Text Word] OR chronic[Text Word] OR persistent[Text Word] OR relapse[Text Word] OR remitting[Text Word] OR remission[Text Word] OR residual[Text Word] OR delayed[Text Word] OR prolonged[Text Word] OR extended[Text Word] OR lingering[Text Word] OR permanent[Text Word] OR fluctuation[Text Word] OR multisystem[Text Word] OR "multi system"[Text Word] OR nonrecovery[Text Word] OR "non recovery"[Text Word] OR subacute[Text Word] OR "sub acute"[Text Word] OR lasting[Text Word] OR continuous[Text Word] OR continually[Text Word] OR continuing[Text Word] OR postacute[Text Word] OR "post acute"[Text Word] OR postdischarge[Text Word] OR "post discharge"[Text Word] OR postinfection[Text Word] OR "post infection"[Text Word] OR postviral[Text Word] OR "post viral"[Text Word] OR postvirus[Text Word] OR "post virus"[Text Word] OR "medium term"[Text Word] OR mediumterm[Text Word]) AND (sequela[Text Word] OR sequelae[Text Word] OR illness[Text Word] OR symptom[Text Word] OR sign[Text Word] OR symptoms[Text Word] OR signs[Text Word] OR prognosis[Text Word] OR rehab[Text Word] OR convalescence[Text Word] OR recuperation[Text Word] OR followup[Text Word] OR "follow up"[Text Word] OR features[Text Word]) AND (covid[Text Word] OR coronavirus[Text Word] OR "corona virus"[Text Word] OR coronovirus[Text Word] OR "corono virus"[Text Word] OR coronavirinae[Text Word] OR "corona virinae"[Text Word] OR Cov[Text Word] OR "2019-nCoV"[Text Word] OR "2019nCoV"[Text Word] OR "19- nCoV"[Text Word] OR "19nCoV"[Text Word] OR "nCoV2019"[Text Word] OR omikron[Text Word] OR Omicron[Text Word] OR "B.1.1.529"[Text Word] OR "B11529"[Text Word] OR xbb[Text Word] OR "nCoV-2019"[Text Word] OR "nCoV19"[Text Word] OR "nCoV-19"[Text Word] OR "HCoV-19"[Text Word] OR "HCoV19"[Text Word] OR "HCoV-2019"[Text Word] OR "HCoV2019"[Text Word] OR "2019 novel"[Text Word] OR Ncov[Text Word] OR "n-cov"[Text Word] OR "SARS-CoV-2"[Text Word] OR "SARSCoV-2"[Text Word] OR "SARSCoV2"[Text Word] OR "SARS-CoV2"[Text Word] OR "SARSCov19"[Text Word] OR "SARS-Cov19"[Text Word] OR "SARSCov-19"[Text Word] OR "SARS-Cov-19"[Text Word] OR "SARSCov2019"[Text Word] OR "SARS-Cov2019"[Text Word] OR "SARSCov-2019"[Text Word] OR "SARS-Cov-2019"[Text Word] OR "SARS2"[Text Word] OR "SARS-2"[Text Word] OR "SARScoronavirus2"[Text Word] OR "SARS-coronavirus-2"[Text Word] OR "SARScoronavirus 2"[Text Word] OR "SARS coronavirus2"[Text Word] OR "SARScoronovirus2"[Text Word] OR "SARS-coronovirus-2"[Text Word] OR "SARScoronovirus 2"[Text Word] OR "SARS coronovirus2"[Text Word] OR "severe acute respiratory syndrome"[Text Word] "severe acute respiratory syndrome")) 5,217

8 ((postcovid[Text Word] OR "post covid"[Text Word] OR postcoronavirus[Text Word] OR "postcorona virus"[Text Word] OR "post coronavirus"[Text Word] OR "post corona virus"[Text Word] OR postcoronovirus[Text Word] OR "postcorono virus"[Text Word] OR "post coronovirus"[Text Word] OR "post corono virus"[Text Word] OR postcoronavirinae[Text Word] OR "postcorona virinae"[Text Word] OR "post coronavirinae"[Text Word] OR "post corona virinae"[Text Word] OR postCov[Text Word] OR "post Cov"[Text Word] OR postsars[Text Word] OR "post sars"[Text Word] OR "post severe acute respiratory syndrome"[Text Word] OR postncov[Text Word] OR "post ncov"[Text Word] OR posthcov[Text Word] OR "post hcov"[Text Word]) AND (syndrome[Text Word] OR disorder[Text Word] OR illness[Text Word] OR sickness[Text Word] OR disease[Text Word] OR condition[Text Word] OR symptom[Text Word] OR sign[Text Word] OR symptoms[Text Word] OR signs[Text Word] OR prognosis[Text Word] OR followup[Text Word] OR "follow up"[Text Word] OR features[Text Word] OR comorbidity[Text Word] OR comorbidities[Text Word] OR "co morbidity"[Text Word] OR "co morbidities"[Text Word] OR multimorbidity[Text Word] OR "multi morbidity"[Text Word] OR multimorbidities[Text Word] OR "multi morbidities"[Text Word] OR survivors[Text Word] OR survival[Text Word] OR risk[Text Word] OR risks[Text Word] OR care[Text Word] OR convalescence[Text Word] OR recuperation[Text Word] OR aftercare[Text Word] OR ambulatory[Text Word] OR outpatient[Text Word] OR "out patient"[Text Word] "out patient")) 31

7 (("long term"[Text Word] OR longterm[Text Word] OR "long haul"[Text Word] OR longhaul[Text Word] OR "long tail"[Text Word] OR longtail[Text Word] OR longduration[Text Word] OR "long duration"[Text Word] OR longlasting[Text Word] OR "long lasting"[Text Word] OR longstanding[Text Word] OR "long standing"[Text Word] OR "medium term"[Text Word] OR mediumterm[Text Word] OR "post-virus"[Text Word] OR "post-viral"[Text Word]) AND (covid[Text Word] OR coronavirus[Text Word] OR "corona virus"[Text Word] OR coronovirus[Text Word] OR "corono virus"[Text Word] OR coronavirinae[Text Word] OR "corona virinae"[Text Word] OR Cov[Text Word] OR "2019-nCoV"[Text Word] OR "2019nCoV"[Text Word] OR "19-nCoV"[Text Word] OR "19nCoV"[Text Word] OR "nCoV2019"[Text Word] OR "nCoV-2019"[Text Word] OR "nCoV19"[Text Word] OR "nCoV-19"[Text Word] OR "HCoV-19"[Text Word] OR "HCoV19"[Text Word] OR "HCoV-2019"[Text Word] OR "HCoV2019"[Text Word] OR "2019 novel"[Text Word] OR Ncov[Text Word] OR "n-cov"[Text Word] OR "SARS-CoV-2"[Text Word] OR "SARSCoV-2"[Text Word] OR "SARSCoV2"[Text Word] OR "SARS-CoV2"[Text Word] OR "SARSCov19"[Text Word] OR "SARS-Cov19"[Text Word] OR "SARSCov-19"[Text Word] OR "SARS-Cov-19"[Text Word] OR "SARSCov2019"[Text Word] OR "SARS-Cov2019"[Text Word] OR "SARSCov-2019"[Text Word] OR "SARS-Cov-2019"[Text Word] OR "SARS2"[Text Word] OR "SARS-2"[Text Word] OR omikron[Text Word] OR Omicron[Text Word] OR "B.1.1.529"[Text Word] OR "B11529"[Text Word] OR xbb[Text Word] OR "SARScoronavirus2"[Text Word] OR "SARS-coronavirus-2"[Text Word] OR "SARScoronavirus 2"[Text Word] OR "SARS coronavirus2"[Text Word] OR "SARScoronovirus2"[Text Word] OR "SARS-coronovirus-2"[Text Word] OR "SARScoronovirus 2"[Text Word] OR "SARS coronovirus2"[Text Word] OR "severe acute respiratory syndrome"[Text Word] "severe acute respiratory syndrome")) 2,257

6 ((long[Text Word] OR enduring[Text Word] OR legacy[Text Word] OR slow[Text Word] OR gradual[Text Word] OR protracted[Text Word] OR lengthy[Text Word] OR chronic[Text Word] OR persistent[Text Word] OR relapsing[Text Word] OR remitting[Text Word] OR remission[Text Word] OR residual[Text Word] OR delayed[Text Word] OR prolonged[Text Word] OR extended[Text Word] OR lingering[Text Word] OR permanent[Text Word] OR fluctuating[Text Word] OR sequelae[Text Word] OR multisystem[Text Word] OR "multi system"[Text Word] OR nonrecovery[Text Word] OR "non recovery"[Text Word] OR subacute[Text Word] OR "sub acute"[Text Word] OR lasting[Text Word] OR continuous[Text Word] OR continual[Text Word] OR continuing[Text Word] OR postacute[Text Word] OR "post acute"[Text Word] OR postdischarge[Text Word] OR "post discharge"[Text Word] OR postinfection[Text Word] OR "post infection"[Text Word] OR postviral[Text Word] OR "post viral"[Text Word] OR postvirus[Text Word] OR "post virus"[Text Word] OR "late effect"[Text Word] OR "late effects"[Text Word]) AND (covid[Text Word] OR coronavirus[Text Word] OR "corona virus"[Text Word] OR coronovirus[Text Word] OR "corono virus"[Text Word] OR coronavirinae[Text Word] OR "corona virinae"[Text Word] OR Cov[Text Word] OR "2019-nCoV"[Text Word] OR "2019nCoV"[Text Word] OR "19-nCoV"[Text Word] OR "19nCoV"[Text Word] OR "nCoV2019"[Text Word] OR "nCoV-2019"[Text Word] OR "nCoV19"[Text Word] OR "nCoV-19"[Text Word] OR "HCoV-19"[Text Word] OR "HCoV19"[Text Word] OR "HCoV-2019"[Text Word] OR "HCoV2019"[Text Word] OR "2019 novel"[Text Word] OR Ncov[Text Word] OR "n-cov"[Text Word] OR "SARS-CoV-2"[Text Word] OR "SARSCoV-2"[Text Word] OR "SARSCoV2"[Text Word] OR "SARS-CoV2"[Text Word] OR "SARSCov19"[Text Word] OR "SARS-Cov19"[Text Word] OR "SARSCov-19"[Text Word] OR "SARS-Cov-19"[Text Word] OR "SARSCov2019"[Text Word] OR "SARS-Cov2019"[Text Word] OR "SARSCov-2019"[Text Word] OR "SARS-Cov-2019"[Text Word] OR "SARS2"[Text Word] OR "SARS-2"[Text Word] OR "SARScoronavirus2"[Text Word] OR "SARS-coronavirus-2"[Text Word] OR "SARScoronavirus 2"[Text Word] OR "SARS coronavirus2"[Text Word] OR omikron[Text Word] OR Omicron[Text Word] OR "B.1.1.529"[Text Word] OR "B11529"[Text Word] OR xbb[Text Word] OR "SARScoronovirus2"[Text Word] OR "SARS-coronovirus-2"[Text Word] OR "SARScoronovirus 2"[Text Word] OR "SARS coronovirus2"[Text Word] OR "severe acute respiratory syndrome"[Text Word] "severe acute respiratory syndrome")) 10,487

5 (longcovid[Text Word] OR "long covid"[Text Word] OR longcoronavirus[Text Word] OR "longcorona virus"[Text Word] OR "long coronavirus"[Text Word] OR "long corona virus"[Text Word] OR longcoronovirus[Text Word] OR "longcorono virus"[Text Word] OR "long coronovirus"[Text Word] OR "long corono virus"[Text Word] OR longcoronavirinae[Text Word] OR "longcorona virinae"[Text Word] OR "long coronavirinae"[Text Word] OR "long corona virinae"[Text Word] OR longCov[Text Word] OR "long Cov"[Text Word] OR longsars[Text Word] OR "long sars"[Text Word] OR "long severe acute respiratory syndrome"[Text Word] OR longncov[Text Word] OR "long ncov"[Text Word] OR longhcov[Text Word] OR "long hcov"[Text Word] OR "post-acute covid"[Text Word] OR "post-acute corona"[Text Word] OR "post-acute corono"[Text Word] OR "post-acute cov"[Text Word] OR "post-acute ncov"[Text Word] OR "post-acute sars"[Text Word] OR "post-acute severe respiratory syndrome"[Text Word]) 5,532

4 (("long term"[Text Word] OR longterm[Text Word] OR "long haul"[Text Word] OR longhaul[Text Word] OR "long tail"[Text Word] OR longtail[Text Word] OR longduration[Text Wo1rd] OR "long duration"[Text Word] OR longlasting[Text Word] OR "long lasting"[Text Word] OR longstanding[Text Word] OR "long standing"[Text Word] OR "medium term"[Text Word] OR mediumterm[Text Word] OR "late effect"[Text Word] OR recurrence[Text Word] OR prolonged[Text Word] OR post-viral[Text Word] OR chronic[Text Word] OR postacute[Text Word] OR "post acute"[Text Word] OR persistent[Text Word]) AND (covid[Text Word] OR coronavirus[Text Word] OR "corona virus"[Text Word] OR coronovirus[Text Word] OR "corono virus"[Text Word] OR coronavirinae[Text Word] OR "corona virinae"[Text Word] OR Cov[Text Word] OR "2019-nCoV"[Text Word] OR 2019nCoV[Text Word] OR "19-nCoV"[Text Word] OR "19nCoV"[Text Word] OR "nCoV2019"[Text Word] OR "nCoV-2019"[Text Word] OR "nCoV19"[Text Word] OR "nCoV-19"[Text Word] OR "HCoV-19"[Text Word] OR "HCoV19"[Text Word] OR "HCoV-2019"[Text Word] OR "HCoV2019"[Text Word] OR "2019 novel"[Text Word] OR Ncov[Text Word] OR "n-cov"[Text Word] OR "SARS-CoV-2"[Text Word] OR "SARSCoV-2"[Text Word] OR "SARSCoV2"[Text Word] OR "SARS-CoV2"[Text Word] OR "SARSCov19"[Text Word] OR "SARS-Cov19"[Text Word] OR "SARSCov-19"[Text Word] OR "SARS-Cov-19"[Text Word] OR "SARSCov2019"[Text Word] OR "SARS-Cov2019"[Text Word] OR "SARSCov-2019"[Text Word] OR "SARS-Cov-2019"[Text Word] OR "SARS2"[Text Word] OR "SARS-2"[Text Word] OR omikron[Text Word] OR Omicron[Text Word] OR "B.1.1.529"[Text Word] OR "B11529"[Text Word] OR xbb[Text Word] OR "SARScoronavirus2"[Text Word] OR "SARS-coronavirus-2"[Text Word] OR "SARScoronavirus 2"[Text Word] OR "SARS coronavirus2"[Text Word] OR "SARScoronovirus2"[Text Word] OR "SARS-coronovirus-2"[Text Word] OR "SARScoronovirus 2"[Text Word] OR "SARS coronovirus2"[Text Word] OR "severe acute respiratory syndrome"[Text Word] "severe acute respiratory syndrome")) 6,103

3 ("post acute sequela"[Text Word] OR "post acute sequela"[Text Word]) AND (covid[Text Word] OR coronavirus[Text Word] OR coronovirus[Text Word] OR "corona virus"[Text Word] OR COV[Text Word] OR omikron[Text Word] OR Omicron[Text Word] OR "B.1.1.529"[Text Word] OR "B11529"[Text Word] OR xbb[Text Word]) 19

2 (PASC[Text Word] OR "chronic covid syndrome"[Text Word]) 888

1 "Post-Acute COVID-19 Syndrome"[Mesh:NoExp] 2,902

***The PubMed strategy was updated on 1.2.24 (8 records) and 1.3.24 (8 records).***

*COVID facet based on terms from:*

World Health Organization (26 May 2021) WHO COVID-19 Database Search Strategy. Systematic search of the COVID-19 literature performed Monday through Friday for the WHO Database. Search strategy as of 26 May 2021. Searches performed by Tomas Allen, Kavita Kothari, and Martha Knuth. Available from: <https://www.who.int/docs/default-source/coronaviruse/who-covid-19-database/who-covid-19_sources_searchstrategy_20210526.pdf?sfvrsn=65209cc2_5>

Canadian Agency for Drugs and Technologies in Health (2.9.21) CADTH COVID-19 Search Strings: COVID-19 — EMBASE (Internet). Available from: <https://covid.cadth.ca/literature-searching-tools/cadth-covid-19-search-strings/>

NICE (18 December 2020) [accessed 17.8.21] COVID-19 rapid guideline: managing the long-term effects of COVID-19 [NG188]. Search history record [PDF]. NICE: London. Available from: <https://www.nice.org.uk/guidance/ng188/evidence/search-strategies-pdf-8957634445>

*PubMed limit:*

Duffy S, de Kock S, Misso K, Noake C, Ross J, Stirk L. Supplementary searches of PubMed to improve currency of MEDLINE and MEDLINE In-Process searches via Ovid. J Med Libr Assoc. 2016 Oct;104(4):309-312. doi: 10.3163/1536-5050.104.4.011. <https://www.ncbi.nlm.nih.gov/pmc/articles/PMC5079494/>

**Europe PMC, including MedRxiv and bioRxiv preprints (Internet): up to 2024/01/24**

**Searched 24.1.24**

**<https://europepmc.org/>**

| **Search terms (limited to preprints only)** | **Results** |
| --- | --- |
| "long-term" OR "long haul" OR "long tail" OR "long duration" OR "long lasting" OR "long standing" OR "medium term" OR "late effects" OR "prolonged" OR "persistent" OR "chronic" OR "post viral" OR "post acute"  AND  "COVID-19" OR "SARS-CoV-2" OR "coronavirus" OR "COVID" OR "NCOV" OR "omicron" OR "Omicron" OR "B.1.1.529" OR "B11529" OR "xbb"  AND  "immunised" OR "immunise" OR "immunisation" OR "immunisations" OR "immunized" OR "immunize" OR "immunization" OR "immunizations" OR "immunity" OR "vaccine" OR "vaccines" OR "vaccination" OR "vaccinations" OR "vaccinated" OR "jab" OR "jabs" OR "shot" OR "shots" OR "booster" OR "boosters" OR "revaccination" OR "revaccinations" OR "revaccinated" OR "unvaccinated"  *All fields* | **17** |
| "COVID-19" OR "SARS-CoV-2" OR "coronavirus" OR "COVID" OR "NCOV" OR omicron OR Omicron OR "B.1.1.529" OR "B11529" OR "xbb"  AND  "sequelae" OR "sickness"  AND  "immunised" OR "immunise" OR "immunisation" OR "immunisations" OR "immunized" OR "immunize" OR "immunization" OR "immunizations" OR "immunity" OR "vaccine" OR "vaccines" OR "vaccination" OR "vaccinations" OR "vaccinated" OR "jab" OR "jabs" OR "shot" OR "shots" OR "booster" OR "boosters" OR "revaccination" OR "revaccinations" OR "revaccinated" OR "unvaccinated"  *All fields* | **2** |
| "PASC" OR "chronic covid syndrome"  AND  "immunised" OR "immunise" OR "immunisation" OR "immunisations" OR "immunized" OR "immunize" OR "immunization" OR "immunizations" OR "immunity" OR "vaccine" OR "vaccines" OR "vaccination" OR "vaccinations" OR "vaccinated" OR "jab" OR "jabs" OR "shot" OR "shots" OR  "booster" OR "boosters" OR "revaccination" OR "revaccinations" OR "revaccinated" OR "unvaccinated" | **0** |
| "post acute sequela" OR "post acute sequela"  AND  (covid OR coronavirus OR coronovirus OR "corona virus" OR COV OR omicron OR Omicron OR "B.1.1.529" OR "B11529" OR "xbb"  AND  "immunised" OR "immunise" OR "immunisation" OR "immunisations" OR "immunized" OR "immunize" OR "immunization" OR "immunizations" OR "immunity" OR "vaccine" OR "vaccines" OR "vaccination" OR "vaccinations" OR "vaccinated" OR "jab" OR "jabs" OR "shot" OR "shots" OR "booster" OR "boosters" OR "revaccination" OR "revaccinations" OR "revaccinated" OR "unvaccinated" | **0** |
| **Total (including duplicates)** | **19** |

***The Europe PMC strategy was updated on 1.2.24 (0 records) and 1.3.24 (0 records).***

**Latin American and Caribbean Health Sciences Literature (LILACS) (Internet): 2022-2024/01/25**

**Searched 25.1.24**

<https://search.bvsalud.org/portal/?lang=en>

Searched Title/Abstract/Subject

Limited to 2022-2024/01/25

Limited to LILACS only

| **Search terms** | **Results** |
| --- | --- |
| ("long-term" OR "long haul" OR "long tail" OR "long duration" OR "long lasting" OR "long standing" OR "medium term" OR "late effects" OR "prolonged" OR "persistent" OR "chronic" OR "post viral" OR "post acute")  TITLE, ABSTRACT, SUBJECT  AND  ("COVID-19" OR "SARS-CoV-2" OR "coronavirus" OR "COVID" OR "NCOV" OR "omicron" OR "Omicron" OR "B.1.1.529" OR "B11529" OR "xbb")  AND  ("immunised" OR "immunise" OR "immunisation" OR "immunisations" OR "immunized" OR "immunize" OR "immunization" OR "immunizations" OR "immunity" OR "vaccine" OR "vaccines" OR "vaccination" OR "vaccinations" OR "vaccinated" OR "jab" OR "jabs" OR "shot" OR "shots" OR "booster" OR "boosters" OR "revaccination" OR "revaccinations" OR "revaccinated" OR "unvaccinated") | 63 |
| ("PASC" OR "chronic covid syndrome")  AND  ("immunised" OR "immunise" OR "immunisation" OR "immunisations" OR "immunized" OR "immunize" OR "immunization" OR "immunizations" OR "immunity" OR "vaccine" OR "vaccines" OR "vaccination" OR "vaccinations" OR "vaccinated" OR "jab" OR "jabs" OR "shot" OR "shots" OR "booster" OR "boosters" OR "revaccination" OR "revaccinations" OR "revaccinated" OR "unvaccinated") | 0 |
| ("post acute sequela" OR "post acute sequela") AND ("covid" OR "coronavirus" OR "coronavirus" OR "corona virus" OR COV OR "omicron" OR "Omicron" OR "B.1.1.529" OR "B11529" OR "xbb")  AND  ("immunised" OR "immunise" OR "immunisation" OR "immunisations" OR "immunized" OR "immunize" OR "immunization" OR "immunizations" OR "immunity" OR "vaccine" OR "vaccines" OR "vaccination" OR "vaccinations" OR "vaccinated" OR "jab" OR "jabs" OR "shot" OR "shots" OR "booster" OR "boosters" OR "revaccination" OR "revaccinations" OR "revaccinated" OR "unvaccinated") | 0 |
| **Total (including duplicates)** | **63** |

***The LILACS strategy was updated on 1.2.24 (0 records) and 1.3.24 (0 records).***

**Cochrane COVID-19 Study Register (www): 2022-2024/01/25**

**Searched 25.1.24**

<https://covid-19.cochrane.org/>

Limited to preprints only

Limited 2022-2024/01/25

| **Search terms** | **Results** |
| --- | --- |
| ("long-term" OR "long haul" OR "long tail" OR "long duration" OR "long lasting" OR "long standing" OR "medium term" OR "late effects" OR "prolonged" OR "persistent" OR "chronic" OR "post viral" OR "post acute")  AND  ("COVID-19" OR "SARS-CoV-2" OR "coronavirus" OR "COVID" OR "NCOV" OR "omicron" OR "Omicron" OR "B.1.1.529" OR "B11529" OR "xbb")  ("immunised" OR "immunise" OR "immunisation" OR "immunisations" OR "immunized" OR "immunize" OR "immunization" OR "immunizations" OR "immunity" OR "vaccine" OR "vaccines" OR "vaccination" OR "vaccinations" OR "vaccinated" OR "jab" OR "jabs" OR "shot" OR "shots" OR "booster" OR "boosters" OR "revaccination" OR "revaccinations" OR "revaccinated" OR "unvaccinated")  ***Limited to preprints*** | 227 (325 refs) |
| ("PASC" OR "chronic covid syndrome")  AND  ("immunised" OR "immunise" OR "immunisation" OR "immunisations" OR "immunized" OR "immunize" OR "immunization" OR "immunizations" OR "immunity" OR "vaccine" OR "vaccines" OR "vaccination" OR "vaccinations" OR "vaccinated" OR "jab" OR "jabs" OR "shot" OR "shots" OR "booster" OR "boosters" OR "revaccination" OR "revaccinations" OR "revaccinated" OR "unvaccinated")  ***Limited to preprints*** | 6 (9 refs) |
| ("post acute sequela" OR "post acute sequela")  AND  ("immunised" OR "immunise" OR "immunisation" OR "immunisations" OR "immunized" OR "immunize" OR "immunization" OR "immunizations" OR "immunity" OR "vaccine" OR "vaccines" OR "vaccination" OR "vaccinations" OR "vaccinated" OR "jab" OR "jabs" OR "shot" OR "shots" OR "booster" OR "boosters" OR "revaccination" OR "revaccinations" OR "revaccinated" OR "unvaccinated")  ***Limited to preprints*** | 0 |
| **Total (including duplicates)** | **233 (334**  **refs)** |

***The Cochrane COVID-19 Study Register strategy was updated on 1.2.24 (0 records) and 1.3.24 (0 records).***

**WHO COVID-19 (Internet): 2022-2024/01/24**

**Searched 24.1.24**

[**https://search.bvsalud.org/global-literature-on-novel-coronavirus-2019-ncov/?lang=en**](https://search.bvsalud.org/global-literature-on-novel-coronavirus-2019-ncov/?lang=en)

| **Search terms** | **Results** |
| --- | --- |
| ("long-term" OR "long haul" OR "long tail" OR "long duration" OR "long lasting" OR "long standing" OR "medium term" OR "late effects" OR "prolonged" OR "persistent" OR "chronic" OR "post viral" OR "post acute")  TITLE, ABSTRACT, SUBJECT  AND  ("COVID-19" OR "SARS-CoV-2" OR "coronavirus" OR "COVID" OR "NCOV" OR "omicron" OR "Omicron" OR "B.1.1.529" OR "B11529" OR "xbb")  AND  ("immunised" OR "immunise" OR "immunisation" OR "immunisations" OR "immunized" OR "immunize" OR "immunization" OR "immunizations" OR "immunity" OR "vaccine" OR "vaccines" OR "vaccination" OR "vaccinations" OR "vaccinated" OR "jab" OR "jabs" OR "shot" OR "shots" OR "booster" OR "boosters" OR "revaccination" OR "revaccinations" OR "revaccinated" OR "unvaccinated")  ***Limited to preprints*** | 674 |
| ("PASC" OR "chronic covid syndrome")  AND  ("immunised" OR "immunise" OR "immunisation" OR "immunisations" OR "immunized" OR "immunize" OR "immunization" OR "immunizations" OR "immunity" OR "vaccine" OR "vaccines" OR "vaccination" OR "vaccinations" OR "vaccinated" OR "jab" OR "jabs" OR "shot" OR "shots" OR "booster" OR "boosters" OR "revaccination" OR "revaccinations" OR "revaccinated" OR "unvaccinated")  ***Limited to preprints*** | 25 |
| ("post acute sequela" OR "post acute sequela") AND ("covid" OR "coronavirus" OR "coronavirus" OR "corona virus" OR COV OR "omicron" OR "Omicron" OR "B.1.1.529" OR "B11529" OR "xbb")  AND  ("immunised" OR "immunise" OR "immunisation" OR "immunisations" OR "immunized" OR "immunize" OR "immunization" OR "immunizations" OR "immunity" OR "vaccine" OR "vaccines" OR "vaccination" OR "vaccinations" OR "vaccinated" OR "jab" OR "jabs" OR "shot" OR "shots" OR "booster" OR "boosters" OR "revaccination" OR "revaccinations" OR "revaccinated" OR "unvaccinated")  ***Limited to preprints*** | 0 |
| **Total (including duplicates)** | **699** |

***** WHO COVID-19 database ceased in June 2023, therefore update searches were not necessary.

- 1. **Data extraction**

Data extracted from the identified studies included the following:

- Study characteristics: country of study, study design, and data source.
- Participant characteristics: population, N Omicron infections, Omicron infection period, subvariants reported, vaccination doses and details, severity of acute infection, previous infection, and participant age, sex, and ethnicity.
- Long COVID outcomes: definition of long COVID, diagnosis of long COVID (e.g., self-reported, average number of long COVID symptoms, prevalence of long COVID symptoms, and risk of long COVID development).
  1. **Meta-analysis**

**1.3.1 Feasibility assessment**

The feasibility assessment determined whether it was possible to conduct pair-wise analyses of the risk of developing long COVID in those who had contracted the Omicron variant of COVID-19 after receiving a vaccine and/or booster dose compared with those who were unvaccinated from the studies retrieved. This assessment determines how comparable the studies are in terms of population, design, and outcomes assessed.

**1.3.2 Conversion of non-odds ratios to odds ratios**

Hazard ratios and relative risk ratios were converted to odds ratios, as a) not all studies reporting odds ratios included data required to convert odds ratios to relative risk, and b) all studies reporting non-odds ratios had low event rates (<10%) and relatively short follow-up time periods^1^.

Hazard ratios were converted to relative risk using equation 1^1^:

$$RR = \frac{1-e^{HR\times\ln\left( 1-P_{0} \right)}}{P_{0}}$$

Relative risk ratios were converted to odds ratios using equation 2^2^:

$$OR = \frac{RR\times\left( 1-P_{0} \right)}{1-\left( RR\times P_{0} \right)}$$

In both equations, *P*_0_ = risk of long COVID in the unvaccinated group.

Original hazard ratios, and the relative risks and odds ratios calculated are shown below:

| **Study** | ***P*_0_** | **Hazard ratio** | **Relative risk** | **Odds ratio** |
| --- | --- | --- | --- | --- |
| Hammel 2023^3^ | 0.049 | 0.82 (0.79, 0.86) | 0.82 (0.79, 0.86) | 0.82 (0.79, 0.86) |
| Hammel 2023^3^ | 0.049 | 0.72 (0.68, 0.76) | 0.72 (0.69, 0.77) | 0.71 (0.67, 0.76) |
| Lundberg-Morris 2023^4^ | 0.006 | 0.59 (0.50, 0.69) | 0.59 (0.50, 0.69) | 0.59 (0.50, 0.69) |
| Wander 2023^5^ | 0.060 | 0.80 (0.78, 0.83) | 0.81 (0.79, 0.83) | 0.80 (0.77, 0.83) |
| Wander 2023^5^ | 0.060 | 0.66 (0.64, 0.69) | 0.67 (0.65, 0.70) | 0.65 (0.63, 0.68) |
| Wu 2024^6^ | 0.068 | - | 0.91 (0.69, 1.19) | 0.90 (0.67, 1.21) |

**1.3.3 Sensitivity analyses**

The following sensitivity analyses were conducted to determine the impact of the addition or removal of specific studies on the pooled risk of long COVID development:

- Leave-one-out analysis: exclusion of a single study at a time to assess the impact of its removal on the main analysis results.
- Substitution analysis replacing the vaccinated (primary course) vs unvaccinated estimate in three studies with their vaccinated (booster dose) vs unvaccinated^3,5,7^.
- Exclusion of adolescent and children-only studies: removal of all studies where the population consisted entirely of adults^6,8,9^.
- Exclusion of pre-print studies: removal of those studies only available as pre-prints at the time of the review’s data extraction^6^.
- Exclusion of potentially overlapping populations: Data sources for all studies were compared; participant inclusion characteristics and time period for those studies with the same data sources were then compared to determine whether it was possible for any participants to be included in both studies. The study with the smaller population was excluded in this sensitivity analysis^3^.
- Exclusion of studies with high hospitalization for acute COVID illness: removal of any studies with >5% hospitalization reported^5,10^.
- Exclusion of unadjusted results: removal of studies that only reported unadjusted risks^10^.

1. **Supplementary Results**
   1. **Meta-analyses feasibility assessment**

Eleven studies were excluded as they did not report risk outcomes^11-21^. One study was excluded from the analyses as this included only liver transplant patients^22^.Three studies were excluded as they reported on the risk of specific symptoms of COVID that were not comparable with other studies^23-25^. Two studies were excluded as they had less than 20 individuals in a comparison group, as such small samples increase uncertainty^26-28^. Three studies reported outcomes that were not reported by enough studies to perform a meta-analysis (three or more studies)^29-31^. None of the studies included in the meta-analyses were considered high risk of bias.

- 1. **Supplementary figures and tables**

**Supplementary Fig. 1:** **Egger’s test funnel plot for “any vaccination” (main analysis)**


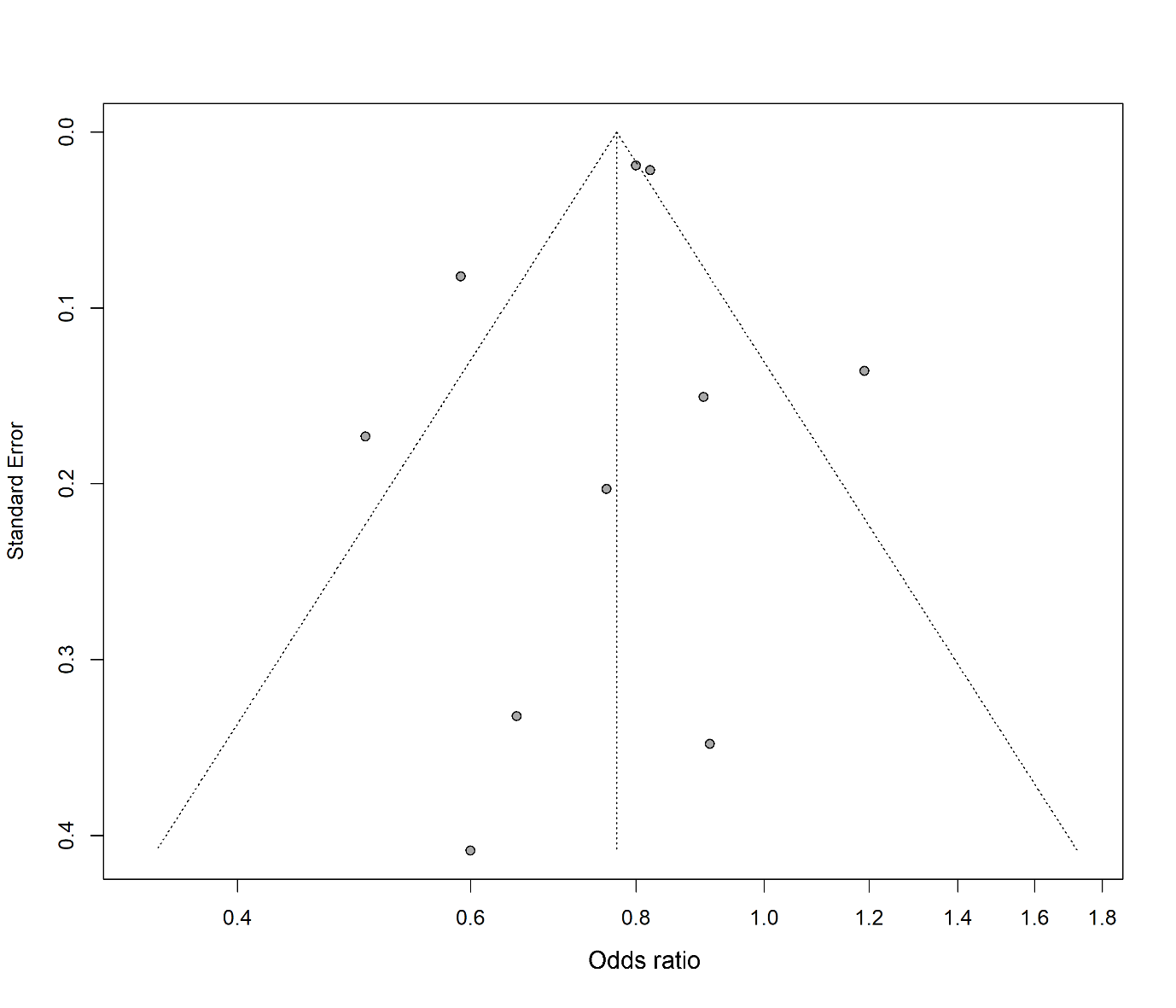


The figure illustrates the Egger’s test funnel plot for the “any vaccination versus no vaccination” meta-analysis (main analysis). In the funnel plot, the central dotted line represents the overall effect, the vertical lines represent the 95% CI, and the dots represent the individual studies. A symmetrical funnel plot indicates a low likelihood of publication bias.

**Supplementary Fig. 2: Egger’s test funnel plot for “any vaccination”**
**(sensitivity analysis substituting primary course estimates for booster dose estimates in three studies)**


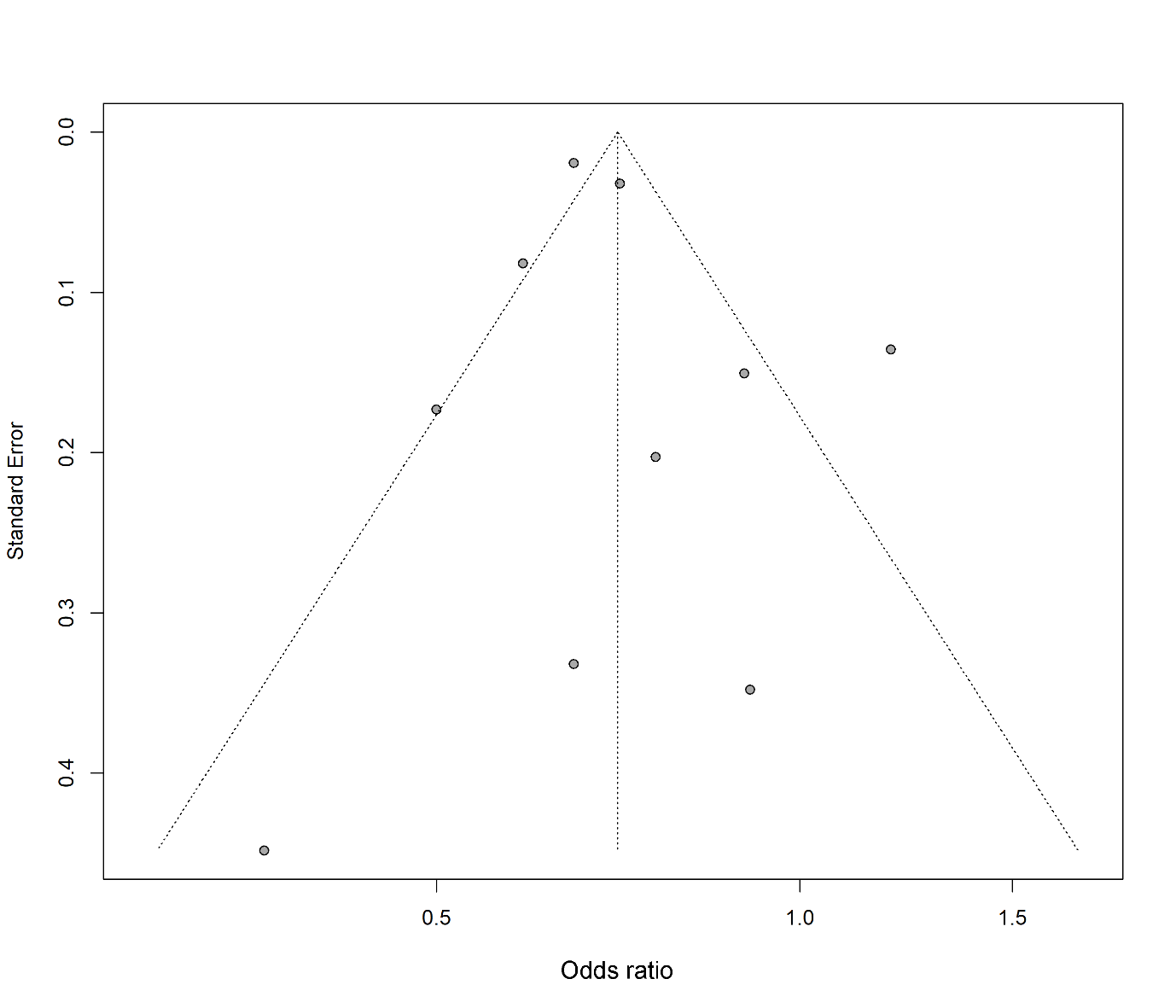


The figure illustrates the Egger’s test funnel plot for the “any vaccination versus no vaccination” sensitivity analysis that substitutes estimates for risk after primary course vaccination with estimates for risk after booster dose vaccination in three studies (Di Fusco 2023, Hammel 2023, Wander 2023). In the funnel plot, the central dotted line represents the overall effect, the vertical lines represent the 95% CI, and the dots represent the individual studies. A symmetrical funnel plot indicates a low likelihood of publication bias.

**Supplementary Fig. 3: Sensitivity analysis for the effect of “any vaccination” on the risk of long COVID compared to unvaccinated (main analysis)**
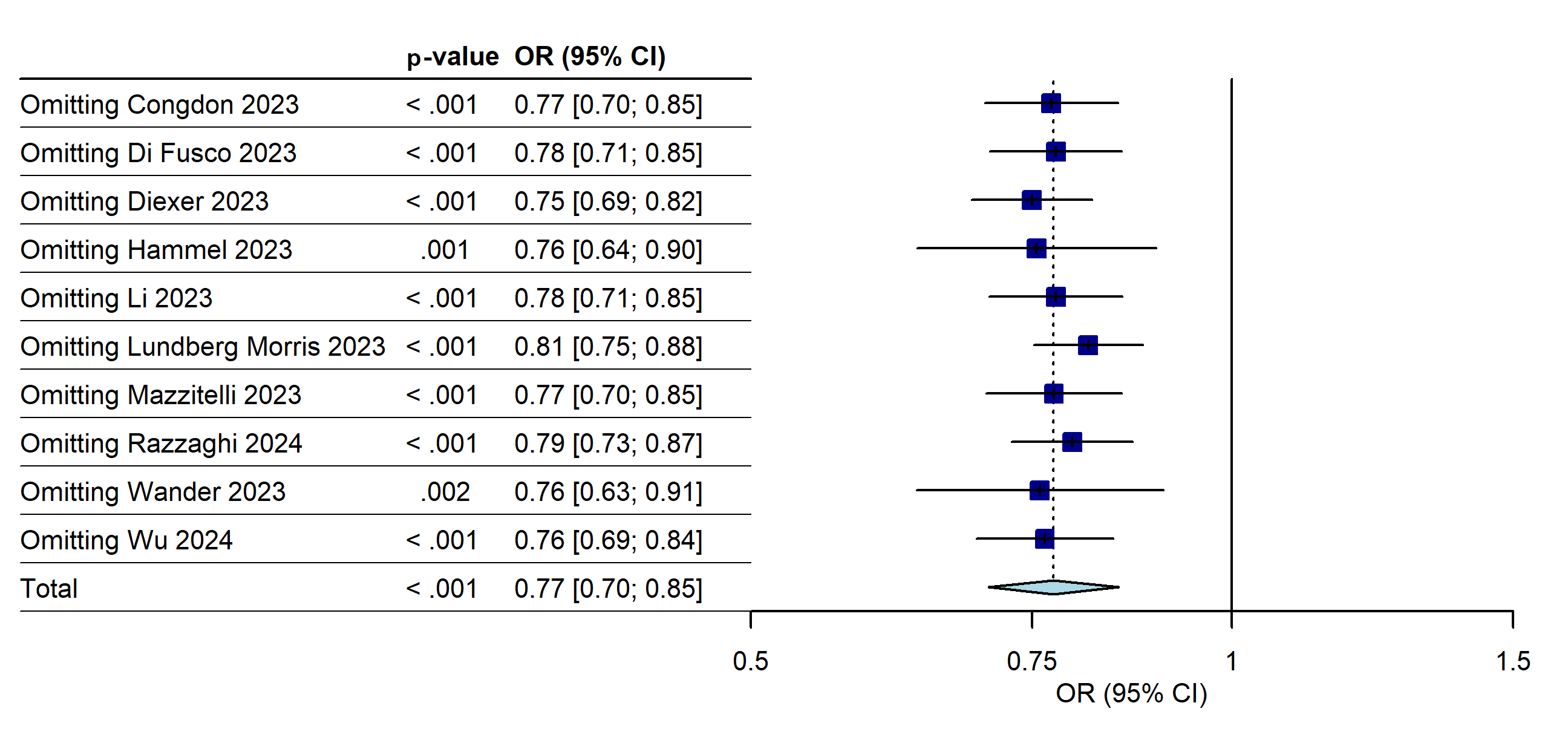


The figure illustrates the leave-one-out sensitivity analysis for the “any vaccination” versus no vaccination meta-analysis (main analysis). Random effects models were used, and all tests were 2-sided. The pooled ORs with each study individually removed are shown as squares, and error bars indicating 95% confidence intervals around the OR. The diamond represents the pooled effect size of the main analysis (all studies) with its 95% confidence interval.

CI, confidence interval; OR, odds ratio**.**

**Supplementary Fig. 4: Sensitivity analysis forest plot for the effect of “any vaccination” on the risk of long COVID compared to unvaccinated (sensitivity analysis substituting primary course estimates for booster dose estimates in three studies)**


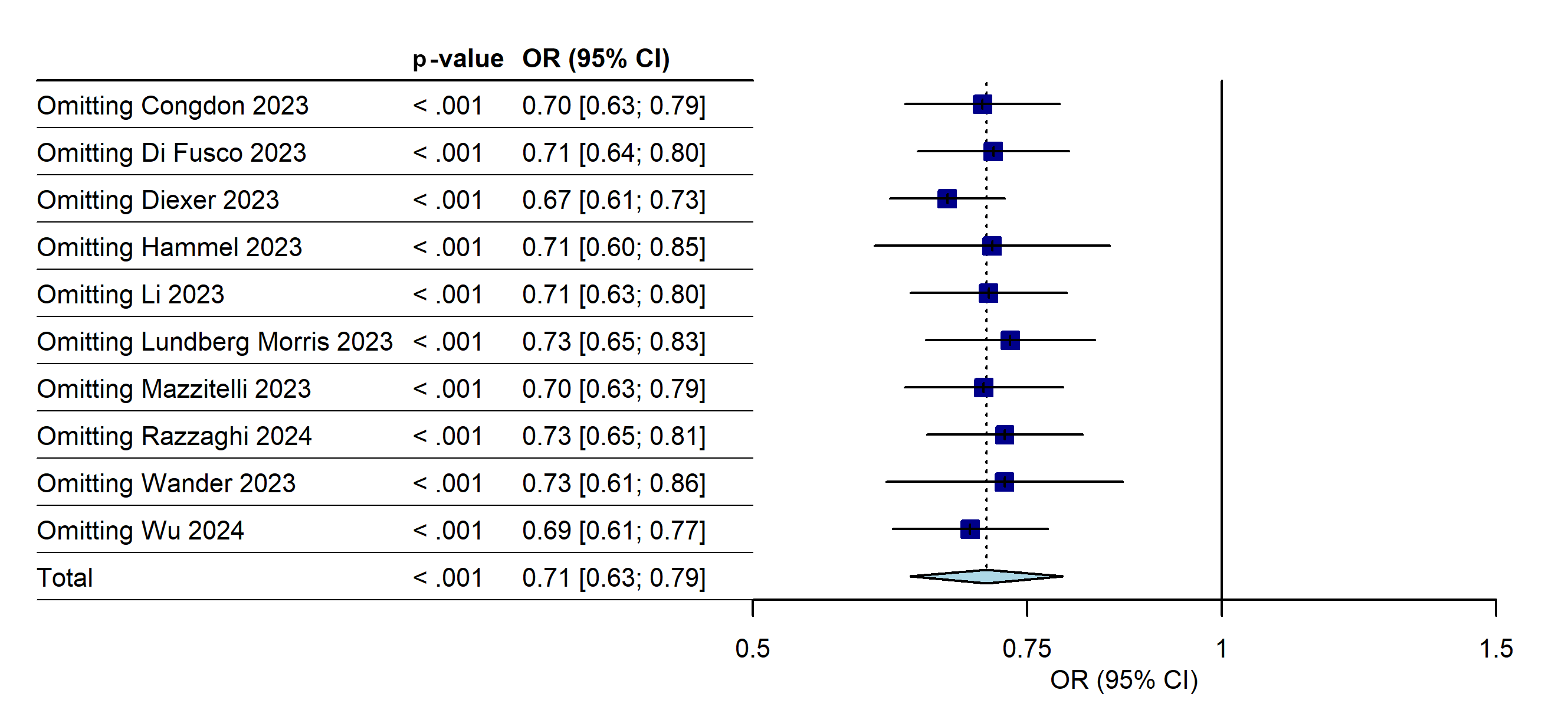


The figure illustrates the leave-one-out sensitivity analysis for the “any vaccination” versus no vaccination meta-analysis for the sensitivity analysis that substitutes estimates for risk after primary course vaccination with estimates for risk after booster dose vaccination in three studies (Di Fusco 2023, Hammel 2023, Wander 2023). Random effects models were used, and all tests were 2-sided. The pooled ORs with each study individually removed are shown as squares, and error bars indicating 95% confidence intervals around the OR. The diamond represents the pooled effect size of the main analysis (all studies) with its 95% confidence interval.

CI, confidence interval; OR, odds ratio.

**Supplementary Fig. 5:** **Sensitivity analysis forest plot for the effect of booster vaccination on the risk of long COVID compared unvaccinated**


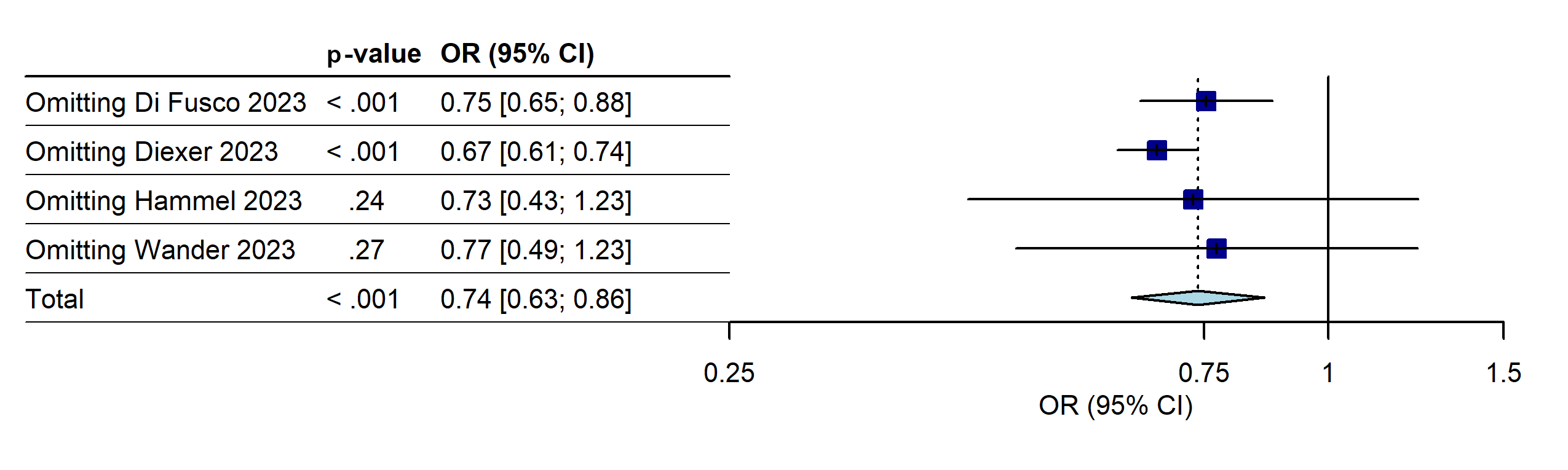


The figure illustrates the leave-one-out sensitivity analysis for the booster vaccination versus no vaccination meta-analysis. Random effects models were used, and all tests were 2-sided. The pooled ORs with each study individually removed are shown as squares, and error bars indicating 95% confidence intervals around the OR. The diamond represents the pooled effect size of the main analysis (all studies) with its 95% confidence interval.

CI, confidence interval; OR, odds ratio.

**Supplementary** **Table 1.** **Characteristics of studies included in the systematic literature review**

| **Study** | **Country** | **Data source** | **Population** | **Study design** | **n participants (Omicron-infected)^a^** | **Omicron subvariants reported** | **Outcomes reported** | **Vaccination groups compared** | **Risk of bias** |
| --- | --- | --- | --- | --- | --- | --- | --- | --- | --- |
| AlBahrani 2023^26^ | Saudi Arabia | Two medical centers | Healthcare workers (adults) | Cross-sectional | 243 | NR | Risk of long COVID | Dose numbers | High |
| Congdon 2023^32^ | USA | Montefiore Medical Center and Montefiore Medical Group | Adult patients meeting criteria to be prescribed nirmatrelvir/ritonavir (18+) | Retrospective | 500 | NR | Risk of long COVID | Vaccinated, unvaccinated | Low |
| Brown 2023^29^ | Australia | Queensland Department of Health’s Notifiable Conditions System | Adult population-based cohort (18+) | Prospective | 2,195 | NR | Risk of long COVID | Booster dose, no booster dose | Medium |
| Gallant 2023^27^ | Canada | Biobanque Québécoise de la COVID-19 (BQC19) | Adult population-based cohorts | Prospective | 290 | NR | Risk of long COVID | Dose numbers | Low |
| Li 2023^9^ | Singapore | Online survey | Children and young people (0–18) | Cross-sectional | 471 | BA.1, BA.2, BA.4, BA.5, XBB | Risk of long COVID | Vaccinated, unvaccinated | Low |
| Hammel 2023^3^ | USA | US Veterans Affairs health care system | Military veterans (adults) | Retrospective | 245,857^b^ | NR | Risk of long COVID | Booster dose, primary course, unvaccinated | Medium |
| Mazzitelli 2023^10^ | Italy | Infectious and Tropical Disease Unit of Padua University Hospital, Padua | Adult outpatients | Retrospective | 681 | NR | Risk of long COVID | Vaccinated, unvaccinated | Low |
| Razzaghi 2024^8^ | USA | Electronic health record data from 41 US health systems | Children (5 to <18) | Retrospective | 289 | NR | Risk of long COVID | Vaccinated, unvaccinated | Low |
| Antonelli 2023^33^ | UK | COVID Symptom Study | Adult population-based cohort (18+) | Case-control | 15,625 | NR | Prevalence and risk of long COVID | Booster, primary course | Low |
| Diexer 2023^34^ | Germany | DigiHero (population-based cohort study on digital health research) | Adult population-based cohort (18+) | Prospective | 11,560 | NR | Prevalence and risk of long COVID | Booster dose, primary course, unvaccinated | Low |
| Herting 2023^22^ | Germany | Liver transplant center | Adult liver transplant patients (18+) | Prospective | 80 | BA.1, BA.2, BA.4, BA.5 | Prevalence and risk of long COVID | Booster dose, primary course | Medium |
| Lundberg-Morris 2023^4^ | Sweden | Swedish healthcare registries | Adult population-based cohort (18+) | Retrospective | 260,390 | NR | Prevalence and risk of long COVID | Vaccinated, unvaccinated | Low |
| Mikolajczyk 2023^31^ | Germany | German National Cohort (NAKO Gesundheltsstudie) | Adult population-based cohort (20+) | Cross-sectional | 45,760 | NR | Prevalence and risk of long COVID (stratified by previous COVID-19 infection and no previous COVID-19 infection) | Additional (4^th^) dose, booster dose, primary course, unvaccinated | Low |
| Woldegiorgis 2023^30^ | Australia | Western Australia Department of Health | Adult population-based cohort (18+) | Prospective | 11,697 | NR | Prevalence and risk of long COVID | Additional (4+) doses, booster dose, no booster dose (0–2 doses) | Low |
| Wu 2024^6^ | USA | National Institute of Health Researching COVID to Enhance Recovery (RECOVER) Initiative | Children (5–11) and adolescents (12–20) | Retrospective | 273,629 | NR | Incidence rate per 10,000 person-week and risk of long COVID and symptom clusters | Vaccinated vs unvaccinated | Medium |
| Wander 2023^5^ | USA | US Veterans Affairs | Military veterans (adults) | Retrospective | 388,980 | NR | Prevalence, cumulative incidence per 100 persons, and risk of long COVID | Booster dose, primary course, unvaccinated | Medium |
| Di Fusco 2023^7^ | USA | Consumer Value Stores (CVS) Health United States test sites | Adult outpatients (18+) | Prospective | 328 | NR | Average number of long COVID symptoms, prevalence and risk of Long COVID and individual long COVID symptoms | Booster dose, primary course, unvaccinated | Medium |
| Ballouz 2023^11^ | Switzerland | Corona Immunitas seroprevalence study and Zurich SARS-CoV-2 Cohort | Adult population-based cohorts (18+) | Prospective | 206 | NR | Prevalence of long COVID | Vaccinated, unvaccinated | Low |
| Cortellini 2023^13^ | Belgium, France, Germany, Italy, Spain, UK | OnCovid registry | Adult cancer patients (18+) | Retrospective | 256 | NR | Prevalence of long COVID | Vaccinated, unvaccinated | Low |
| Hedberg 2023^16^ | Sweden | SmiNet, Stockholm Regional Healthcare Data Warehouse, Statistics Sweden, the National Vaccination Register, and the Swedish Intensive Care Registry | Adult population-based cohort (18+) | Retrospective | 215,279 | NR | Prevalence of long COVID | Vaccinated, unvaccinated | Low |
| Reme 2023^19^ | Norway | Norwegian Emergency Preparedness Register | Adult population-based cohort (30-70) | Prospective | 90,695 | NR | Prevalence of long COVID | Vaccinated, unvaccinated | Low |
| Thaweethai 2023^21^ | USA | Participants recruited from 85 sites across the United States | Adult population-based cohort (18+) | Prospective | 4,897 | NR | Prevalence of long COVID | Primary course, unvaccinated | Low |
| de Bruijn 2023^12^ | Netherlands | Long COVID-study | Adult population-based cohort (18+) | Prospective | 3,823 | NR | Prevalence of long COVID and individual long COVID symptoms | Booster dose, primary course | Low |
| Domenech-Montoliu 2023^15^ | Spain | Valencia Region Vaccine Information System | Adult population-based cohorts (18-64) | Retrospective | 134 | BA.1, BA.2 | Average number of long COVID symptoms and prevalence of long COVID | Booster dose, primary course | Low |
| Nehme 2023^18^ | Switzerland | The CoviCare program | Adult outpatient cohort | Prospective | 1,807 | NR | Prevalence of long COVID and individual long COVID symptoms | Vaccinated, unvaccinated | Low |
| Huh 2024^23^ | South Korea | A combined COVID-19 database constructed by the Korea Disease Control and Prevention Agency and the National Health Insurance Service of the Republic of Korea | Adult population-based cohort (18+) | Retrospective | 7,998,854 | BA.1, BA.2 | Prevalence and risk of post-COVID conditions | Booster dose, primary course, unvaccinated | Low |
| Di Fusco 2024^14^ | USA | Retail pharmacy testing locations | Adult long COVID outpatients (18+) | Prospective | 505 | XBB dominant | Average number of long COVID symptoms, prevalence and risk of ≥2 and ≥3 symptoms, and prevalence and risk of individual long COVID symptoms | Booster dose, unvaccinated (or past the time of assumed vaccine-induced immunity) | Medium |
| Kahlert 2023^17^ | Switzerland | Healthcare networks | Adult healthcare workers (adults) | Prospective | 963 | BA.1 | Average number of long COVID symptoms | Booster dose, primary course, unvaccinated | Low |
| Richard 2023^25^ | USA | Voluntary recruits (active-duty military) | Active duty US military health system beneficiaries (adults) | Prospective | 853 | NR | Risk of individual long COVID symptoms | Booster dose, primary course | Low |
| Spiliopoulos 2023^24^ | Denmark | Danish Nationwide Questionnaire Study (EFTER-COVID) | Adult population-based cohort (15+) | Prospective | 13,724 | NR | Prevalence and risk of individual long COVID symptoms | Booster dose, primary course | Low |
| Sun 2023^20^ | USA | Consumer Value Stores Health United States test sites | Adult outpatients (18+) | Prospective | 328 | NR | Latent class analysis of data from Di Fusco 2023 | Booster dose, primary course, unvaccinated | Medium |

NR, not reported; UK, United Kingdom; USA, United States of America; vs, versus.
^a^The n value of Omicron-infected individuals is listed if available; otherwise, the total N across different variants is listed; ^b^Total number of delta- and Omicron-infected individuals.

**Supplementary table 2. Study outcomes and outcome definitions**

| **Study** | **Long COVID outcome** | **Long COVID assessment time points** | **Definition reported** |
| --- | --- | --- | --- |
| AlBahrani 2023^26^ | Long COVID | 3 months | - Symptoms that last for at least 2 months and cannot be explained by an alternative diagnosis |
| Antonelli 2023^33^ | Long COVID | 1 month, 3 months | - Persisting pre-specified symptoms |
| Ballouz 2023^11^ | Long COVID | 6 months | - Post COVID condition-related symptoms |
| Brown 2023^29^ | Long COVID | 3 months | - Ongoing symptoms |
| Congdon 2023^32^ | Long COVID | 4 months | - Any of eleven listed common long COVID symptoms |
| Cortellini 2023^13^ | Long COVID | 1 month | - Any ongoing symptom or instrumental abnormality detected between 4 weeks and 12 weeks after the start of acute SARS-CoV-2 infection and that could not be explained by an alternative diagnosis |
| de Bruijn 2023^12^ | Long COVID | 3 months | - At least one of the five significantly elevated symptoms in long COVID: Fatigue, dyspnoea, difficulties with a busy environment, problems with memory, brain fog |
|  | Symptoms | 3 months | - Fatigue, dyspnoea, difficulties with a busy environment, problems with memory, brain fog |
| Diexer 2023^34^ | Long COVID | 3 months | - The presence of any symptom in the time window ≥12 weeks after infection (24 listed in questionnaire) |
| Di Fusco 2023^7^ | Long COVID | 1 month | - ≥3 symptoms of 20 pre-specified symptoms |
| Di Fusco 2024^14^ | Long COVID severity | 1 month | - ≥3 symptoms of 30 pre-specified symptoms - ≥2 symptoms |
|  | Symptoms | 1 month | - General symptoms e.g. tiredness or fatigue - Respiratory and cardio symptoms e.g. Difficulty breathing - Neurologic symptoms e.g. difficulty thinking - Digestive/other symptoms e.g. Diarrhoea |
| Domenech-Montoliu 2023^15^ | Long COVID | 3 months | - Symptoms for at least two months and three months from the date of a previous SARS-CoV-2 infection |
| Gallant 2023^27^ | Long COVID | 1 month | - Persistent symptoms from a 28-symptom questionnaire. |
| Hammel 2023^3^ | Long COVID | 1 month | - ICD 10 codes including: U.09.9, U07.1, Z86.16, and/or J12.82 |
| Hedberg 2023^16^ | Long COVID | 3 months | - U.09.9 code given by any healthcare profession in primary care outpatient specialist care, or inpatient care |
| Herting 2023^22^ | Long COVID | 1 to >3 months | - Long-lasting symptoms |
| Huh 2024^23^ | Post-COVID conditions | 1 month | - List of several conditions and diseases |
| Kahlert 2023^17^ | Long COVID | 3 months | - Post-acute sequelae of SARS-CoV-2 symptom score |
| Li 2023^9^ | Long COVID | 3 months | - One or more persistent physical symptoms for a minimum duration of 3 months with an impact on everyday functioning |
| Lundberg-Morris 2023^4^ | Long COVID | 1 month | - U09.9 as the main or secondary diagnosis |
| Mazzitelli 2023^10^ | Long COVID | 1 month, 3 months | - Post-acute COVID-19 based on literature definitions^53^ |
| Mikolajczyk 2023^31^ | Long COVID | 4 months | - Presence of post-COVID condition (from list of 21 symptoms) |
| Nehme 2023^18^ | Long COVID | 3 months | - The presence of ≥1 of the symptoms listed. Only symptoms with a new onset after the test date were taken into consideration. |
|  | Symptoms | 3 months | - Fatigue, concentration difficulties, headache, insomnia, smell issues, taste issues, dyspnoea, cough, myalgia, arthralgia, paraesthesia, chest pain, palpitations, digestive issues, hair loss |
| Razzaghi 2024^8^ | Long COVID | 1 month | - Symptom-based or diagnosed long COVID (a single diagnosis code fell within the case definition for probable long COVID) - Diagnosed long COVID only (two or more healthcare visits with diagnosis codes specific for long COVID) |
| Reme 2023^19^ | Long COVID | 3 months | - ICPC-2 code (R992) with at least one code for a persistent symptom, for example fatigue or pain |
| Richard 2023^25^ | Symptoms | 1 month | - New or increased difficulty exercising - New or increased difficulty doing daily activities (like walking or going up stairs) |
| Spiliopoulos 2023^24^ | Symptoms | 4 months | - Borderline or above depression (Hospital Anxiety and Depression Scale) - Borderline or above anxiety (Hospital Anxiety and Depression Scale) - Substantial fatigue (Fatigue Assessment Scale) - Cognitive Complaints in Bipolar Disorder Rating Assessment |
| Sun 2023^20^ | Symptom burden | 1 month | - Latent classes of post-COVID Symptoms |
| Thaweethai 2023^21^ | Long COVID | 6 months | - Post-acute sequalae of COVID |
|  | Symptom clusters | 6 months | - Participants classified as PASC positive were clustered into subgroups using unsupervised learning including symptoms identified with LASSO. Symptoms highly correlated with those identified by LASSO were reported. |
| Wander 2023^5^ | Long COVID | 1 month | - ICD-10 code U09.9 for post–COVID-19 condition, unspecified |
| Woldegiorgis 2023^30^ | Long COVID | 3 months | - New or ongoing COVID-19 illness-related symptoms or health issues |
| Wu 2024^6^ | Long COVID | 1 month | - Conclusive or probable post-acute sequalae of COVID (A single healthcare visit indicating a diagnosis of PASC or MIS or the presence of a documented SARS-CoV-2 infection alongside a minimum of two long-COVID-compatible diagnoses) |
|  | Symptom clusters | 1 month | - Respiratory cluster - Musculoskeletal cluster - Cardiac cluster - Syndrome cluster - Gastrointestinal cluster |

PASC, post-acute sequelae of SARS-CoV-19; MIS, multisystem inflammatory syndrome.

**Supplementary** **Table 3.** **Quality assessment NOS scores of included studies (excluding cross-sectional studies)**

| **Author/year** | **Selection**  **(max 4 stars)** | | **Comparability  (max 2 stars)** | | **Outcome**  **(max 3 stars)** | | **Total**  **(out of 9 stars)** | **Risk of bias** |
| --- | --- | --- | --- | --- | --- | --- | --- | --- |
| **Prospective** | | | | | | | | |
| Di Fusco 2023^7^ | 3 | | 1 | | 1 | | 5 | Medium |
| Sun 2023^20^ | 3 | | 1 | | 1 | | 5 | Medium |
| Ballouz 2023^11^ | 4 | | 2 | | 1 | | 7 | Low |
| Gallant 2023^27^ | 4 | | 2 | | 3 | | 9 | Low |
| Herting 2023^22^ | 3 | | 2 | | 1 | | 6 | Medium |
| Kahlert 2023^17^ | 3 | | 2 | | 2 | | 7 | Low |
| Reme 2023^19^ | 3 | | 2 | | 3 | | 9 | Low |
| Spiliopoulos 2023^24^ | 4 | | 2 | | 2 | | 8 | Low |
| Woldegiorgis 2023^30^ | 4 | | 2 | | 2 | | 8 | Low |
| Diexer 2023^34^ | 4 | | 1 | | 2 | | 7 | Low |
| de Bruijn 2023^12^ | 3 | | 2 | | 3 | | 8 | Low |
| Thaweethai 2023^21^ | 4 | | 1 | | 2 | | 7 | Low |
| Nehme 2023^18^ | 4 | | 2 | | 2 | | 8 | Low |
| Brown 2023^29^ | 4 | | 1 | | 1 | | 6 | Medium |
| Richard 2023^25^ | 4 | | 2 | | 2 | | 8 | Low |
| Antonelli 2023^33^ | 3 | | 2 | | 2 | | 7 | Low |
| Di Fusco 2024^14^ | 3 | | 2 | | 1 | | 6 | Medium |
| **Retrospective** | | | | | | | | |
| Domenech-Montoliu 2023^15^ | 3 | 2 | | 2 | | 7 | | Low |
| Mazzitelli 2023^10^ | 4 | 1 | | 2 | | 7 | | Low |
| Razzaghi 2024^8^ | 3 | 2 | | 2 | | 7 | | Low |
| Hammel 2023^3^ | 3 | 1 | | 2 | | 6 | | Medium |
| Congdon 2023^32^ | 4 | 2 | | 1 | | 7 | | Low |
| Hedberg 2023^16^ | 4 | 2 | | 2 | | 8 | | Low |
| Lundberg-Morris 2023^4^ | 4 | 2 | | 2 | | 8 | | Low |
| Wander 2023^5^ | 1 | 2 | | 2 | | 6 | | Medium |
| Wu 2024^6^ | 2 | 2 | | 2 | | 6 | | Medium |
| Cortellini 2023^13^ | 3 | 2 | | 2 | | 7 | | Low |
| Huh 2024^23^ | 4 | 2 | | 2 | | 8 | | Low |

NOS, Newcastle-Ottawa Scale.

**Supplementary Table 4. Classification of vaccination status by this review, and the number of doses received, minimum time between last vaccine dose received and SARS-CoV-2 infection, time to SARS-CoV-2 infection from last vaccine dose, and vaccine type received, where reported by included studies**

| **Study** | **Population** | **Vaccination status and doses reported** | **Minimum time between last vaccine dose and SARS-CoV-2 infection** | **Time to SARS-CoV-2 infection from last vaccine dose** | **Vaccine type** |
| --- | --- | --- | --- | --- | --- |
| AlBahrani 2023^26^ | Adults | **Vaccinated:**  4 doses: 12 (4.9%)  3 doses: 223 (91.8%)  2 doses: 5 (2.1%)  **Unvaccinated**:  0 doses: 3 (1.2%) | NR | NR | NR (assumed mixed) |
| Antonelli 2023^33^ | Adults | **Booster dose**  3 doses: 7,984 (51.1%)  **Primary course**  2 doses: 7,641 (48.9%) | 7 days | NR | NR (assumed mixed) |
| Ballouz 2023^11^ | Adults | **Vaccinated**  3 doses: 59 (19.3%)  1-2 doses: 173 (56.7%)  **Unvaccinated**  0 doses: 72 (24.0%) | NR | - <6 months: 180 (77.6%) - ≥6 months: 52 (22.4%)     *(mixed wild-type, Delta, and Omicron population)* | mRNA: BNT162b2, mRNA-1273 (1 patient [0.4%] received Ad26.COV2.S) |
| Brown 2023^29^ | Adults | **Booster dose**  ≥3 doses: 1,220 (83.2%)  **No booster dose**  ≤2 doses: 975 (16.8%) | NR | ≥6 months since last dose: 338 (23.1%) | NR (assumed mixed) |
| de Bruijn 2023^12^ | Adults | **Booster dose**  ≥1 dose after primary course: 2,970 (76.1%)  **Primary course**  2 doses (BNT162b2, mRNA-1273, ChAdOx1) or 1 dose^a^: (Ad26.COV2.S): 853 (21.8%)  **Unvaccinated**  Partially vaccinated^a^: 21 (0.5%)  Unvaccinated: 60 (1.5%) | 14 days (BNT162b2, mRNA-1273, ChAdOx1), 28 days (Ad26.COV2.S), and 7 days (any booster dose) | NR | Mixed: BNT162b2, mRNA-1273, ChAdOx1, Ad26.COV2.S |
| Congdon 2023^32^ | Adults | **Vaccinated**  Booster dose^b^: 338 (67.6%)  Primary course^b^: 123 (24.6%)  **Unvaccinated**  Unvaccinated^b^: 39 (7.8%) | NR | NR | Mixed: BNT162b2, mRNA-1273, Ad26.COV2.S, ChAdOx1 |
| Cortellini 2023^13^ | Adults | **Vaccinated**  ≥2 doses: 196 (86.0%)  **Unvaccinated**  ≤1 dose: 32 (14.0%) | NR | NR | Mixed: BNT162b2, mRNA-1273, ChAd0x1, Ad26.COV2.S   - Booster dose:   - BNT162b2: 67 (49.3%)   - mRNA-1273: 36 (26.5%)   - Ad.26.COV2.S: 1 (0.7%)   - ChAdOx-2: 24 (17.6%)   - Not specified: 8 (5.9%) - Primary course   - BNT162b2: 77 (42.1%)   - mRNA-1273: 40 (21.9%)   - Ad.26.COV2.S: 4 (2.2%)   - ChAdOx-2: 39 (21.3%)   - Not specified: 23 (12.6%) - Partially vaccinated:   - BNT162b2: 23 (35.4%)   - mRNA-1273: 17 (26.2%)   - ChAdOx-2: 12 (18.5%)   - Not specified: 13 (20.0%) |
| Di Fusco 2023^7^ | Adults | **Booster dose**  3 doses^c^: 87 (26.5%)  **Primary course**  2 doses^c^: 86 (26.2%)  **Unvaccinated**  0 doses: 155 (47.3%) | 14 days | - Boosted: mean 2.3 months (SD: 1.9) - Primed: mean 6.9 months (SD: 3.0) | BNT162b2 |
| Di Fusco 2024^14^ | Adults | **Booster dose**  Bivalent booster dose: 260 (51.5%)  **Unvaccinated**^d^  Unvaccinated or monovalent dose >12 months before enrolment^b^: 245 (48.5%) | NR | Time to week 4 after positive test (start of long covid):   - All participants: mean 337 days (SD 209) - BNT162b2-vaccinated: mean 165 days (SD 46) - Not up to date^4^: mean 546 days (SD 121) | Bivalent BNT162b2 BA.4/5 |
| Diexer 2023^34^ | Adults | **Booster dose**  ≥3 doses: 8,780 (71.3%)  **Primary course**  1-2 doses: 2,199 (17.9%)  **Unvaccinated**  0 doses: 1,328 10.8% | NR | NR | NR (assumed mixed) |
| Domenech-Montoliu 2023^15^ | Adults | **Booster dose**  3 doses: 90 (76.9%)  **Primary course**  1-2 doses: 27 (23.1%) | 14 days | NR | NR (assumed mixed) |
| Gallant 2023^27^ | Adults | **Vaccinated**  ≥2 doses: 280 (96.6%)  **Unvaccinated**  ≤1 dose: 10 (3.4%) | NR | NR | mRNA: BNT162b2, mRNA-1273 |
| Hammel 2023^3^ | Adults | **Booster dose**  3 doses (or 2 if Ad26.COV2.S: NR for Omicron  **Primary course**  2 doses (or 1 if Ad26.COV2.S: NR for Omicron  **Unvaccinated**  ≤2 doses: NR for Omicron | 7 days | NR | Mixed: BNT162b2, mRNA-1273, Ad26.COV2.S |
| Hedberg 2023^16^ | Adults | **Vaccinated**  2 doses: 123,289 (70.9%)  3 doses: 23,829 (13.7%)  **Unvaccinated**  1 dose: 5,044 (2.9%)  0 doses: 21,757 (12.5%) | NR | NR | NR (assumed mixed) |
| Herting 2023^22^ | Adults | **Booster dose**  3 doses: 59 (60.2%)  4 doses: 25 (25.6%)  5 doses: 2 (2%)  **Primary course**  2 doses: 12 (12.2%) | NR | - Median 130 days (IQR 88.8–183.3) | Mixed: mRNA and/or vector-based vaccine |
| Huh 2024^23^ | Adults | **Booster dose**  3 doses: 5,001,039 (62.5%)  **Primary course**  2 doses: 2,603,042 (32.5%)  **Unvaccinated**  0 doses: 394,773 (4.9%) | NR | NR | Mixed: BNT162b2, mRNA-1273, ChAd0x1 |
| Kahlert 2023^17^ | Adults | **Booster dose and primary course**  ≥3 doses (booster dose): NR  1-2 doses (primary course): NR  Vaccinated (booster and primary): 876 (91.0%)  **Unvaccinated**  0 doses: 84 (8.7%) | 7 days | NR | Mixed: BNT162b2, mRNA-1273, Other   - >99% either mRNA-1273 or BNT162b2 |
| Li 2023^9^ | Children | **Vaccinated**  ≥2 doses: 205 (43.5%)  **Unvaccinated**  <2 doses: 266 (56.5%) | 2 weeks | NR | mRNA (2 patients [0.4%] received Sinovac) |
| Lundberg-Morris 2023^4^ | Adults | **Vaccinated**  1-5 doses: 224,330 (86.2%)  **Unvaccinated**  0 doses: 36,060 (13.8%) | NR. To avoid double registrations of one vaccine dose, required at least 19 days between doses for BNT162b2 and at least 25 days for ChAd0x1 and mRNA-1283 | - Median 126 days (IQR 47-160)     *(Includes non-Omicron variants)* | Mixed: BNT16b2, mRNA-1273, ChAd0x1 |
| Mazzitelli 2023^10^ | Adults | **Vaccinated**  Doses NR: 238 (34.9%)  **Unvaccinated**  Doses NR: 443 (65.1%) | NR | NR | NR (assumed mixed) |
| Mikolajczyk 2023^31^ | Adults | **Additional booster dose**  4 doses: NR for Omicron  **Booster dose**  3 doses: NR for Omicron  **Primary course**  1-2 doses: NR for Omicron  **Unvaccinated**  0 doses: NR for Omicron | NR | NR | NR (assumed mixed) |
| Nehme 2023^18^ | Adults | **Vaccinated**  2-3 doses: 1338 (74.0%)  **Unvaccinated**  0 doses: 287 (15.9%)  1 dose: 107 (5.9%) | NR | NR | mRNA: BNT162b2, mRNA-1273 |
| Razzaghi 2024^8^ | Adolescents | **Vaccinated**  ≥1 dose: NR for Omicron  **Unvaccinated**  0 doses: NR for Omicron | 28 days | NR | mRNA |
| Reme 2023^19^ | Adults | **Vaccinated**  Vaccinated: 87,975 (97%)  **Unvaccinated**  Unvaccinated: 6,349 (7%) | NR | NR | NR (assumed mixed) |
| Richard 2023^25^ | Adults | **Booster dose**  ≥1 dose after primary course: NR for Omicron  **Primary course**  ≥2 doses (BNT162b2, mRNA-1273) or ≥1 dose (Ad26.COV2.S): NR for Omicron  **Unvaccinated**  0 or ≤1 dose (BNT162b2, mRNA-1273): NR for Omicron | 14 days | NR | Mixed: BNT162b2, mRNA-1273, Ad26.COV2.S |
| Spiliopoulos 2023^24^ | Adults (15+ years) | **Booster dose**  3 doses: 9,355 (57.5%)  **Primary course**  2 doses: 6,240 (38.4%)  **Unvaccinated**  1 dose: 92 (0.6%)  0 doses: 574 (3.5%) | 14 days | NR | Mixed: BNT162b2, mRNA-1273, ChAd0x1, Ad26.COV2.S |
| Sun 2023^20^ | Adults | **Booster dose**  ≥1 after primary course: 87 (26.5%)  **Primary course**  Primary course: 86 (26.2%)  **Unvaccinated**  No evidence of vaccination: 155 (47.3%) | NR | NR | BNT162b2 |
| Thaweethai 2023^21^ | Adults | **Primary course**  Vaccinated: 4,224 (93.0%)  **Unvaccinated**  Unvaccinated: 318 (7.0%) | NR | NR | NR (assumed mixed) |
| Wander 2023^5^ | Adults | **Booster dose**  ≥3 (BNT162b2, mRNA-1273) or ≥2 (Ad26.COV2.S) doses: 104 236 (26.8%)  **Primary course**  2 doses (BNT162b2, mRNA-1273) or 1 dose (Ad26.COV2.S): 134 603 (34.6%)  **Unvaccinated**  None: 104 236 (26.8%) | NR | NR | Mixed: BNT162b2, mRNA-1273, Ad26.COV2.S |
| Woldegiorgis 2023^30^ | Adults | **Additional booster dose**  ≥4 doses: 3,748 (16.6%)  **Booster dose**  3 doses: 17,186 (75.8%)  **No booster dose**  0-2 doses: 1,810 (7.7%) | 1 week | NR | NR (assumed mixed) |
| Wu 2024^6^ | Adolescents (12-20 years) | **Vaccinated**  ≥1 dose: 139,001 (50.8%)  **Unvaccinated**  0 doses: 134,628 (49.2%) | 14 days | NR | BNT162b2 |

NR, not reported.

^a^Additionally, a primary course was defined as completed when a participant had a self-reported or confirmed SARS-CoV-2 infection more than 8 weeks prior to receiving one dose which itself was at least 14 days prior to a positive or enrolment. Participants were partially vaccinated if they received a single dose of the two-dose vaccines or if they tested positive or enrolled within the immunization period. Participants were unvaccinated if they had received no dose at baseline. A participant was considered as ‘boostered’ when the primary course was completed and an additional dose was administered at least 7 days prior to a positive test or enrolment.

^b^Exact dose numbers not reported.

^c^For self-reported immunocompromised patients, 3 doses for primary course and 4 doses for booster dose.

^d^“unvaccinated/not-up-to-date” cohort: either (1) did not report receipt of any COVID-19 vaccine before testing, or (2) reported having received their last original monovalent dose >12 months before enrolment, considered to be past the time of assumed vaccine-induced immunity.

Where only participant numbers were reported, percentages were calculated by reviewers.

**Supplementary Table 5. Summary of long COVID outcomes reported by included studies**

| **Outcome reported** | **Number of studies** | **Studies** | **Overall summary** |
| --- | --- | --- | --- |
| Mean number of long COVID symptoms | 5 | Di Fusco 2023, Di Fusco 2024, Domenech-Montoliu 2023, Kahlert 2023, Sun 2023 | Most studies reported that primary course and booster vaccination was associated with a lower number of symptoms compared with no vaccination, and that booster doses were associated with a lower number of symptoms compared with just primary course vaccination. |
| Prevalence or incidence of long COVID | 18 | Antonelli 2023, Ballouz 2023, de Bruijn 2023, Cortellini 2023, Diexer 2023, Di Fusco 2023, Domenech-Montoliu 2023, Hedberg 2023, Huh 2024, Lundberg-Morris 2023, Mikolajczyk 2023, Nehme 2023, Reme 2023, Spiliopoulos 2023, Thaweethai 2023, Wander 2023, Woldegiorgis 2023, Wu 2024 | The majority of studies reported that long COVID prevalence and incidence were lower in those receiving vaccination and further doses (booster, additional dose) compared with no vaccination or vaccination with a lower number of doses. |
| Risk of long COVID development | 20 | AlBahrani 2023, Antonelli 2023, Brown 2023, Congdon 2023, Diexer 2023, Di Fusco 2023, Gallant 2023, Hammel 2023, Herting 2023, Huh 2024, Li 2023, Lundberg-Morris 2023, Mazzitelli 2023, Mikolajczyk 2023, Razzaghi 2024, Richard 2023, Spiliopoulos 2023, Wander 2023, Woldegiorgis 2023, Wu 2024 | Overall, primary course vaccination, booster vaccination, and additional vaccine doses were associated with a numerically or statistically significantly lower risk of long COVID compared with either no vaccination or vaccination with a lower number of doses. |

**Supplementary Table 6. Reported average number of long COVID symptoms**

| **Study** | **Vaccine** | **Time from infection to long COVID assessment** | **Vaccine dose** | **N participants** | **Mean (SD) number of symptoms** | **Comparison** | **p value** |
| --- | --- | --- | --- | --- | --- | --- | --- |
| **Omicron-infected patients** | | | | | | | |
| Di Fusco 2023^7^ | BNT162b2 | 1 month | Booster dose | 87 | 2.0 (2.3) | Between all | <0.01 |
|  |  |  | Primary course | 86 | 3.1 (3.5) |  |  |
|  |  |  | Unvaccinated | 155 | 3.7 (4.1) |  |  |
|  |  | 3 months | Booster dose | 73 | 1.4 (1.9) | Between all | <0.01 |
|  |  |  | Primary course | 77 | 2.8 (3.5) |  |  |
|  |  |  | Unvaccinated | 142 | 3.3 (4.0) |  |  |
|  |  | 6 months | Booster dose | 67 | 1.1 (1.8) | Between all | <0.001 |
|  |  |  | Primary course | 72 | 2.8 (3.6) |  |  |
|  |  |  | Unvaccinated | 121 | 3.4 (4.2) |  |  |
| Domenech-Montoliu 2023^15^ | NR (mixed) | 3 months | Booster dose | 90 | 1.5 (2.55) | Between both | NR |
|  |  |  | Primary course | 27 | 2.4 (3.7) |  |  |
| Kahlert 2023^17^ | Mixed | 3 months | Booster dose | 727 | 0.49 (95% CI, 0.41–0.58) | Unvaccinated | 0.295 |
|  |  |  | Primary course | 242 | 0.71 (95% CI, 0.53–0.95) | Unvaccinated | 0.028 |
|  |  |  | Unvaccinated | 102 | 0.36 (95% CI, 0.22–0.60) | NA | NA |
| Sun 2023^20^ | BNT162b2 | Up to 6 months | Booster dose | NR | NR (data in figure) | None | NA |
|  |  |  | Primary course | NR |  |  |  |
|  |  |  | Unvaccinated | NR |  |  |  |
| **Long COVID patients only** | | | | | | | |
| Di Fusco 2024^14^ | BNT162b2 (bivalent) | 1 month | Booster dose | 260 | 2.4 (3.0) | Between both | 0.006 |
|  |  |  | Unvaccinated | 245 | 2.9 (3.2) |  |  |
|  |  | 3 months | Booster dose | 244 | 2.1 (2.4) | Between both | 0.028 |
|  |  |  | Unvaccinated | 226 | 2.8 (3.7) |  |  |
|  |  | 6 months | Booster dose | 233 | 2.0 (2.5) | Between both | 0.115 |
|  |  |  | Unvaccinated | 211 | 2.4 (2.7) |  |  |

CI, confidence intervals; NA, not applicable; NR, not reported; SD, standard deviation.

**Supplementary Table 7. Prevalence of ≥3 and ≥2 symptoms reported by Di Fusco 2024**

| **Study** | **Time from infection to long COVID assessment** | **Number of symptoms** | **Prevalence** | | |
| --- | --- | --- | --- | --- | --- |
|  |  |  | **Unvaccinated^a^** | **Booster dose** | **p value** |
| Di Fusco 2024^14^ | 1 month | ≥3 symptoms | 35.5% | 24.2% | 0.006 |
|  | 3 months |  | 27.0% | 21.3% | 0.15 |
|  | 6 months |  | 26.5% | 17.2% | 0.017 |
|  |  | ≥2 symptoms | 37.0% | 25.8% | 0.017 |

^a^Baseline prevalence in that study (least vaccinated group).

**Supplementary Table 8. Prevalence of long COVID after Omicron infection by vaccination status**

| **Study** | **Vaccine(s)** | **Time from infection to long COVID assessment** | **Unvaccinated** | **Primary course** | **Booster dose** | **Additional booster dose** | **Vaccinated (N doses not specified)** | **p value** |
| --- | --- | --- | --- | --- | --- | --- | --- | --- |
| Antonelli 2023^33^ | NR (mixed) | ≥4 weeks | - | 4.9% (NR/NR) | 5.1% (NR) | - | - | NR |
|  |  | ≥12 weeks | - | 0.34% (NR/NR) | 0.27% (NR/NR) | - | - | NR |
| Ballouz 2023^11^ | mRNA | 6 months | Data presented as figure | Data presented as figure | |  | - | NR |
| de Bruijn 2023^12^ | Mixed | 3 months | - | 28.8% (NR/853) | 27.4% (NR/2,970) | - | - | NR |
| Cortellini 2023^13^ | Mixed | ≥4 weeks | 9.4% (3/32) | - | - | - | 6.1% (12/196) | 0.49 |
| Diexer 2023^34^ | NR (mixed) | ≥12 weeks | No previous infection  11.36% (NR/1,093) | No previous infection  16.76% (NR/1,780) | No previous infection  13.17% (NR/8,518) | - | - | NR |
|  |  |  | Previous infection  0.43% (NR/235) | Previous infection  1.94%% (NR/419) | Previous infection  1.56%% (NR/262) | - | - | NR |
| Di Fusco 2023^7^ | BNT162b2 | 1 month | 43.9% (68/155) | 41.9% (36/86) | 29.9% (26/87) | - | - | ≥0.05 |
|  |  | 3 months | 42.3% (60/142) | 41.6% (32/77) | 23.3% (17/73) | - | - | <0.05 |
|  |  | 6 months | 44.6% (54/121) | 37.5% (27/72) | 14.9% (10/67) | - | - | <0.001 |
| Domenech-Montoliu 2023^15^ | NR (mixed) | 3 months | - | 13.3% (12/90) | 29.6% (8/27) | - | - | NR |
| Hedberg 2023^16^ | NR (mixed) | ≥90 days (max 240 days) | 0.20% (104/62,019) | 0.20% (287/147,118) | | - | - | NR |
| Lundberg-Morris 2023^4^ | Mixed | ≥28 days | 0.6% (202/36,060) | 0.3% (727/224,330) | |  |  | NR |
| Mikolajczyk 2023^31^ | NR (mixed) | ≥4 months | No previous infection  29.54% (NR/5,088) | No previous infection  33.04% (NR/7,942) | No previous infection  34.32% (NR/29,249) | No previous infection  23.51% (NR/1,127) | - | NR |
|  |  |  | Previous infection  6.61% (NR/469) | Previous infection  7.16% (NR/1,075) | Previous infection  8.67% (NR/784) | Previous infection  3.85% (NR/26) | - | NR |
| Nehme 2023^18^ | mRNA | 3 months | 18.1% (NR/394) | 9.7% (NR/1,338) | | - | - | 0.001 |
| Reme 2023^19^ | NR (mixed) | ≥3 months | 0.06% (NR/6,348) | - | - | - | 0.12% (NR/84,347) | NR |
| Thaweethai 2023^21^ | NR (mixed) | ≥6 months | Acute cohort (enrolled ≤30 days since infection)  17% (15/86) | Acute cohort (enrolled ≤30 days since infection)  9.7% (195/2,016) | - | - | - | NR |
|  |  |  | Post-acute cohort (enrolled >30 days after infection)  22% (50/232) | Post-acute cohort (enrolled >30 days after infection)  16% (367/2,208) | - | - | - | NR |
| Wander 2023^5^ | Mixed | ≥1 month (max 12 months) | 5.99% (6,244/104,236) | 4.90% (6,596/134,603) | 3.83% (5,747/150,141) | - | - | NR |
| Woldegiorgis 2023^30^ | NR (mixed) | 90 days | 19.9% (140/703) | | 18.5% (1,652/8,919) | 4+ doses  16.2% (337/2,075) | - | NR |

NR, not reported.

**Supplementary Table 9. Long COVID risk outcomes**

| **Study** | **Vaccine(s)** | **Time from infection to long COVID assessment** | **Subgroup** | **Vaccine dose comparison** | | **Measurement** | **Risk (95% CIs)** | **p value** |
| --- | --- | --- | --- | --- | --- | --- | --- | --- |
| **Vaccinated (any no. of doses) vs unvaccinated** | | | | | | | | |
| Congdon 2023^32^ | NR (mixed) | 4 months | - | 2-–3 | 0 | OR (multivariate) | *0.91 (0.46–1.79)* | 0.78 |
| Li 2023^9^ | mRNA | 3 months | - | 2+ | 0 | OR (multivariate) | 0.65 (0.34–1.25) | 0.20 |
| Lundberg-Morris 2023^4^ | Mixed | ≥28 days | - | 1–3 | 0 | HR (partially adjusted) | 0.59 (0.50–0.69) | NR |
|  |  |  |  |  |  | HR (fully adjusted) | 0.59 (0.50–0.69) | <0.001 |
| Mazzitelli 2023^10^ | NR (mixed) | 1 month | - | NR | NR | OR (univariate) | 0.63 (0.44–0.89) | 0.009 |
|  |  | 3 months |  |  |  | OR (univariate) | 0.76 (0.51– 1.13) | 0.175 |
| Razzaghi 2024^8^ | mRNA | ≥1 month | 5–11 yrs; symptom-based or diagnosed long COVID | 1+ | 0 | VE (adjusted) | 23.8% (4.9–39.0) | NR |
|  |  |  | 5–11 yrs; diagnosed long COVID | 1+ | 0 | VE (adjusted) | 48.2% (-20.8–77.8) | NR |
|  |  |  | 12–17 yrs; symptom-based or diagnosed long COVID | 1+ | 0 | VE (adjusted) | 49.6% (29.2–64.1) | NR |
|  |  |  | 5–17 yrs: symptom-based or diagnosed long COVID | 2+ | 0 | VE (adjusted) | 45% (35–53) | NR |
|  |  |  | 5–11 yrs; symptom-based or diagnosed long COVID | 2+ | 0 | VE (adjusted) | Data presented as figure | NR |
|  |  |  | 12–17 yrs; symptom-based or diagnosed long COVID | 2+ | 0 | VE (adjusted) | Data presented as figure | NR |
|  |  |  | 5–17 yrs: diagnosed long COVID | 2+ | 0 | VE (adjusted) | Data presented as figure | NR |
|  |  |  | 5–11 yrs; diagnosed long COVID | 2+ | 0 | VE (adjusted) | Data presented as figure | NR |
|  |  |  | 12–17 yrs; diagnosed long COVID | 2+ | 0 | VE (adjusted) | Data presented as figure | NR |
| Wu 2024^6^ | BNT162b2 | ≥28 days | 5–11 yrs | 1+ | 0 | RR (adjusted) | 1.24 (0.92–1.66) | NR |
|  |  |  | 12–20 yrs | 1+ | 0 | RR (adjusted) | 0.91 (0.69–1.19) | NR |
| **Primary course vs unvaccinated** | | | | | | | | |
| Di Fusco 2023^7^ | BNT162b2 | 6 months | - | 2 | 0 | OR (adjusted) | 0.60 (0.27–1.34) | 0.296 |
| Hammel 2023^3^ | Mixed | ≥4 weeks | - | 2 | 0 | HR (adjusted) | 0.82 (0.79–0.86) | NR |
| Wander 2023^5^ | Mixed | ≥1 month | - | 2 | 0 | HR (adjusted) | 0.80 (0.78–0.83) | NR |
| **Booster dose vs unvaccinated** | | | | | | | | |
| Di Fusco 2023^7^ | BNT162b2 | 6 months | - | 3 | 0 | OR (adjusted) | 0.36 (0.15–0.87) | 0.019 |
| Diexer 2023^34^ | NR (mixed) | ≥12 weeks | - | 3 | 0 | OR (adjusted) | *1.19 (0.92–1.56)* | NR |
| Hammel 2023^3^ | Mixed | ≥4 weeks | - | 3 | 0 | HR (adjusted) | 0.72 (0.68–0.76) | NR |
| Wander 2023^5^ | Mixed | ≥1 month | - | 3 | 0 | HR (adjusted) | 0.66 (0.64–0.69) | NR |
| **Booster dose vs primary course** | | | | | | | | |
| Antonelli 2023^33^ | NR (mixed) | ≥4 weeks | All adults | 3 | 2 | OR (adjusted) | 1.01 (0.85–1.19) | 0.948 |
|  |  |  | Younger adults (18–59 yrs) | 3 | 2 | OR (adjusted) | 0.91 (0.73–1.12) | 0.368 |
|  |  |  | Older adults (60+ yrs) | 3 | 2 | OR (adjusted) | 1.10 (0.83–1.45) | 0.001^b^ |
|  |  | ≥12 weeks | All ages | 3 | 2 | OR (adjusted) | 0.77 (0.39–1.52) | 0.448 |
|  |  |  | Younger adults (18–59 yrs) | 3 | 2 | OR (adjusted) | 0.73 (0.29–1.86) | 0.511 |
| Di Fusco 2023^7^ | BNT162b2 | 6 months | - | 3 | 2 | OR (adjusted) | 0.59 (0.21–1.65) | 0.459 |
| Diexer 2023^34^ | NR (mixed) | ≥12 weeks | - | 3 | 1–2 | OR (adjusted) | *0.78 (0.64–0.93)* | NR |
| Herting 2023^22^ | Mixed | 1 to >3 months | - | 3+ | 2 | OR (multivariate) | 2.94 (0.84–10.28) | 0.085 |
| **Booster dose vs no booster dose** | | | | | | | | |
| Brown 2023^29^ | NR (mixed) | 12 weeks | - | 3+ | 0–2 | OR (multivariate) | 0.73 (0.56–0.96) | 0.02 |
| **Additional booster dose vs no booster dose** | | | | | | | | |
| Woldegiorgis 2023^30^ | NR (mixed) | 90 days | - | 4+ | 0-2 | RR (adjusted) | *0.63 (0.52– 0.77)* | <0.001^a^ |
| **Additional booster dose vs booster dose** | | | | | | | | |
| Mikolajczyk 2023^31^ | NR (mixed) | ≥4 months | - | 4 | 3 | OR (multivariate) | 0.52 (0.41–0.61) | NR |
| Woldegiorgis 2023^30^ | NR (mixed) | 90 days | - | 4+ | 3 | RR (adjusted) | *0.71 (0.63–0.77)* | <0.001^a^ |
| **Increase in dose number** | | | | | | | | |
| AlBahrani 2023^26^ | NR (mixed) | ≥3 months | - | 1 unit increase in dose number | | OR | 0.53 (0.18–1.56) | 0.25 |
| Gallant 2023^27^ | mRNA | 1 month | - | 1 unit increase in dose number | | RR (multivariate) | 0.92 (0.79–1.98) | 0.489 |

CI, confidence interval; HR, hazard ratio; NR, not reported; OR, odds ratio; RR, relative risk; VE, vaccine effectiveness.

Data in italics have had their direction adjusted to match that of other studies (risk of higher number of doses compared with lower number of doses/no doses).

^a^p-value reported for overall comparison of ≥4 doses, 3 doses, and 0–2 doses; ^b^Likely error in publication.

**Supplementary Table 10. Table of data used in the five meta-analyses**

| **Study** | **Vaccine dose comparison in study** | **Odds ratios (95% CIs)**  **included in meta-analysis** |
| --- | --- | --- |
| **Vaccinated vs unvaccinated (main analysis)** | | |
| Congdon 2023^32^ | Vaccinated (2–3 doses) vs no vaccination | 0.91 (0.46–1.79)^a^ |
| Di Fusco 2023^7^ | Primary course vs no vaccination | 0.60 (0.27–1.34) |
| Diexer 2023^34^ | Booster dose (3+) vs no vaccination | 1.19 (0.92–1.56)^a^ |
| Hammel 2023^3^ | Primary course vs no vaccination | 0.82 (0.79–0.86)^b^ |
| Li 2023^9^ | Vaccinated (≥2 doses) vs no vaccination | 0.65 (0.34–1.25) |
| Lundberg-Morris 2023^4^ | Vaccinated (1–5 doses) vs no vaccination | 0.59 (0.50–0.69)^c^ |
| Mazzitelli 2023^10^ | Vaccinated (doses NR) vs no vaccination | 0.76 (0.51–1.13) |
| Razzaghi 2024^8^ | Vaccinated (≥1 dose) vs no vaccination | 0.50 (0.36–0.71)^d^ |
| Wander 2023^5^ | Primary course vs no vaccination | 0.80 (0.77–0.83)^e^ |
| Wu 2024^6^ | Vaccinated (≥1 dose) vs no vaccination | 0.90 (0.67–1.21)^f^ |
| **Vaccinated vs unvaccinated (substitution sensitivity analysis: primary course to booster dose in three studies)** | | |
| Congdon 2023^32^ | Vaccinated (2–3 doses) vs no vaccination | 0.91 (0.46–1.79)^a^ |
| Di Fusco 2023^7^ | Booster dose vs no vaccination | 0.36 (0.15– 0.87) |
| Diexer 2023^34^ | Booster dose vs no vaccination | 1.19 (0.92–1.56)^a^ |
| Hammel 2023^3^ | Booster dose vs no vaccination | 0.71 (0.67–0.76)^g^ |
| Li 2023^9^ | Vaccinated (≥2 doses) vs no vaccination | 0.65 (0.34–1.25) |
| Lundberg-Morris 2023^4^ | Vaccinated (1–5 doses) vs no vaccination | 0.59 (0.50–0.69)^c^ |
| Mazzitelli 2023^10^ | Vaccinated (doses NR) vs no vaccination | 0.76 (0.51–1.13) |
| Razzaghi 2024^8^ | Vaccinated (≥1 dose) vs no vaccination | 0.50 (0.36–0.71)^d^ |
| Wander 2023^5^ | Booster dose vs no vaccination | 0.65 (0.63–0.68)^g^ |
| Wu 2024^6^ | Vaccinated (≥1 dose) vs no vaccination | 0.90 (0.67–1.21)^f^ |
| **Primary course vs unvaccinated** | | |
| Di Fusco 2023^7^ | Primary course vs no vaccination | 0.60 (0.27–1.34) |
| Hammel 2023^3^ | Primary course vs no vaccination | 0.82 (0.79–0.86)^b^ |
| Wander 2023^5^ | Primary course vs no vaccination | 0.80 (0.77– 0.83)^e^ |
| **Booster dose vs unvaccinated** | | |
| Di Fusco 2023^7^ | Booster dose vs no vaccination | 0.36 (0.15–0.87) |
| Diexer 2023^34^ | Booster dose vs no vaccination | 1.19 (0.92–1.56) |
| Hammel 2023^3^ | Booster dose vs no vaccination | 0.71 (0.67–0.76)^g^ |
| Wander 2023^5^ | Booster dose vs no vaccination | 0.65 (0.63–0.68)^h^ |
| **Booster dose vs primary course** | | |
| Antonelli 2023^33^ | Booster dose vs primary course | 0.77 (0.39–1.52) |
| Di Fusco 2023^7^ | Booster dose vs primary course | 0.59 (0.21–1.65) |
| Diexer 2023^34^ | Booster dose vs primary course | 0.78 (0.64–0.93) |

CI, confidence interval; HR, hazard ratio; NR, not reported.

^a^Directionality adjusted by reviewers.

^b^Converted from hazard ratio of 0.82 (0.79–0.86).

^c^Converted from hazard ratio of 0.59 (0.50–0.69).

^d^Converted from vaccine effectiveness of 49.6% (29.2%–64.1%).

^e^Converted from hazard ratio of 0.80 (0.78– 0.83).

^f^Converted from relative risk of 0.91 (0.69–1.19).

^g^Converted from hazard ratio of 0.72 (0.68, 0.76).

^h^Converted from hazard ratio of 0.66 (0.64, 0.69).

**Supplementary Table 11.** **Sensitivity analyses and test of heterogeneity for the effect of “any vaccination” on the risk of long COVID compared with no vaccination (main analysis)**

| **Sensitivity analysis** | **Odds ratio (95% CIs)** | **p value** | **Q** | **Degrees of freedom** | **Q: p value** | **I^2^** |
| --- | --- | --- | --- | --- | --- | --- |
| Exclude potential population overlap | 0.76 (0.64–0.90) | 0.0014 | 30.86 | 8 | 0.0001 | 74.1% |
| Exclude children/adolescents | 0.79 (0.72–0.87) | <0.0001 | 24.13 | 6 | 0.0005 | 75.1% |
| Exclude preprint | 0.76 (0.69–0.84) | <0.0001 | 32.00 | 8 | <0.0001 | 75.0% |
| Exclude high hospitalization rate | 0.76 (0.62–0.92) | 0.0053 | 32.46 | 7 | <0.0001 | 78.4% |
| Exclude unadjusted results | 0.78 (0.71–0.85) | <0.0001 | 32.92 | 8 | <0.0001 | 75.7% |

Random effects models were used, and all tests were two-sided; no adjustments were made for multiple comparisons

CI, confidence interval; I^2^, quantifying heterogeneity; Q, test of heterogeneity.

**Supplementary Table 12.** **Sensitivity analyses and test of heterogeneity for the effect of “any vaccination” on the risk of long COVID compared with no vaccination (sensitivity analysis substituting primary course estimates for booster dose estimates in three studies)**

| **Sensitivity analysis** | **Odds ratio (95% CIs)** | **p value** | **Q** | **Degrees of freedom** | **Q: p value** | **I^2^** |
| --- | --- | --- | --- | --- | --- | --- |
| Exclude potential population overlap | 0.71 (0.60–0.85) | 0.0001 | 31.16 | 8 | 0.0001 | 74.3% |
| Exclude children/adolescents | 0.71 (0.63–0.80) | 0.0001 | 29.00 | 6 | <0.0001 | 79.3% |
| Exclude preprint | 0.69 (0.61–0.77) | <0.0001 | 31.79 | 8 | <0.0001 | 74.8% |
| Exclude high hospitalization rate | 0.72 (0.60–0.87) | 0.0008 | 38.96 | 7 | 0.0001 | 75.8% |
| Exclude unadjusted results | 0.71 (0.64–0.80) | <0.0001 | 37.00 | 8 | <0.0001 | 78.4% |

Random effects models were used, and all tests were two-sided; no adjustments were made for multiple comparisons.

CI, confidence interval; I^2^, quantifying heterogeneity; Q, test of heterogeneity.

**Supplementary** **Table 13. Sensitivity analyses and test of heterogeneity for the effect of any booster vaccination on the risk of long COVID compared with no vaccination (random effects)**

| **Sensitivity analysis** | **Odds ratio (95% CIs)** | **p value** | **Q** | **Degrees of freedom** | **Q: p value** | **I^2^** |
| --- | --- | --- | --- | --- | --- | --- |
| Exclude potential population overlap | 0.73 (0.43–1.23) | 0.2390 | 21.22 | 2 | <0.0001 | 90.6% |

Random effects models were used, and all tests were two-sided; no adjustments were made for multiple comparisons.

CI, confidence interval; I^2^, quantifying heterogeneity; Q, test of heterogeneity.

**Supplementary** **Table 14. GRADE assessment**

| **Meta-analysis** | **N studies** | **Study design** | **Risk of bias** | **Inconsistency** | **Indirectness** | **Imprecision** | **Dissemination bias** | **Other consideration** | **Pooled OR (95% CI)** | **p value** | **Certainty of Evidence (GRADE)** |
| --- | --- | --- | --- | --- | --- | --- | --- | --- | --- | --- | --- |
| **Vaccinated vs unvaccinated (main analysis)** | 10 | Observational | None | Serious^a^ | Serious^d^ | None | None | None | 0.77 (0.70, 0.85) | <0.0001 | ꚚOOO  Very low |
| **Vaccinated vs unvaccinated (substitution sensitivity analysis: primary course to booster dose in three studies)** | 10 | Observational | None | Serious^b^ | Serious^d^ | None | None | None | 0.71 (0.63, 0.79) | <0.0001 | ꚚOOO  Very low |
| **Primary course vs unvaccinated** | 3 | Observational | None | None | Serious^d^ | None | None | None | 0.81 (0.79, 0.83) | <0.0001 | ꚚOOO  Very low |
| **Booster dose vs unvaccinated** | 4 | Observational | None | Serious^c^ | Serious^d^ | None | None | None | 0.74 (0.63, 0.86) | <0.0001 | ꚚOOO  Very low |
| **Booster dose vs primary course** | 3 | Observational | None | None | Serious^d^ | None | None | None | 0.77 (0.65, 0.92) | 0.004 | ꚚOOO  Very low |

Random effects models were used for the meta-analyses pooling ORs, and all tests were two-sided.

CI, confidence interval; GRADE, Grading of Recommendations Assessment, Development, and Evaluation; I^2^, quantifying heterogeneity; N, total number; OR, odds ratio; Q, test of heterogeneity.

"Very low” certainty rating indicates that the true effect is likely to be substantially different from the estimate of effect, due to very serious limitations in the body of evidence.

^a^Lower grading due to substantial heterogeneity, I^2^=72.4%, X^2^=32.59, p(Q)<0.001.

^b^Lower grading due to substantial heterogeneity, I^2^=74.8%, X^2^=35.71, p(Q)<0.001.

^c^Lower grading due to substantial heterogeneity, I^2^=88.2%, X^2^=25.46, p(Q)<0.001.

^d^Lower grading due to inconsistency in long COVID definitions and follow-up periods, different vaccines used, and likely variable time since vaccination.

**Supplementary** **Table 15. Modified NOS questions**

| **Question** | **Decision** | **Stars awarded** |
| --- | --- | --- |
| **Selection (max 4)** | | |
| **1. Representativeness of the exposed cohort [vaccinated/COVID-19 positive]** | a) Truly representative of target population (e.g., nationwide database) | 1 |
|  | b) Somewhat representative of target population (e.g., city, hospital/hospital system, social media survey) | 1 |
|  | c) Selected groups of participants (i.e., by subgroup: sex, race, occupation, insurance coverage, comorbidity, disease severity, ICU treatment status, pre-existing condition). Restricting inclusion criteria to adults does not count as a subgroup | 0 |
|  | d) No description of the derivation of the cohort | 0 |
| **2. Selection of the non-exposed cohort [unvaccinated]** | a) Drawn from the same community/database/hospital as the exposed cohort | 1 |
|  | b) Drawn from a different source | 0 |
|  | c) No description of the derivation of the non-exposed cohort | 0 |
| **3. Ascertainment of exposure [vaccination]** | a) Diagnosis based upon clinical judgment, or record-linkage (e.g., ICD) | 1 |
|  | b) Parental/personal recall only (self-report of vaccination) | 1 |
|  | c) No information | 0 |
| **4. Evaluation of whether outcome of interest was present prior to COVID-19 infection/symptoms were worsened post-infection** | a) Yes | 1 |
|  | b) No | 0 |
| **Comparability (max 2)** | | |
| **1. Comparability of cohorts on the basis of the design or analysis, or statistical consideration of confounders** | a) Controls/adjusts and/or matches and/or regression analysis for both age and sex | 1 |
|  | b) Controls/adjusts and/or matches and/or regression analysis for comorbidities | 1 |
| **Outcome (maximum 3 stars [2 for retrospective])** | | |
| **1. Assessment of outcome [long COVID symptom under investigation]** | a) Validated objective assessment tool (e.g., established cognitive testing or fatigue measurement tool: MoCA, FACIT) for at least one outcome of interest | 1 |
|  | b) Structured/systematic interview or questionnaire conducted by trained healthcare or research professional or ICD 10 code | 1 |
|  | c) Unstructured self-report (i.e., open question regarding symptoms) and/or not conducted by trained healthcare or research professional (i.e., self-administered) or not stated | 0 |
|  | d) No description | 0 |
| **2. Adequacy of follow-up of cohorts (NA for retrospective)** | a) Complete follow-up; all subjects accounted for | 1 |
|  | b) Subjects lost to follow-up unlikely to introduce bias: ≤10% of initial sample size lost, or description provided of those lost | 1 |
|  | c) Lost >10% of initial sample size during follow-up, and no description of those lost | 0 |
|  | d) No statement | 0 |
| **3. Statistical methodology** | a) Statistical test used to analyze the data clearly described, appropriate and measures of association presented including confidence intervals and probability level (p-value) | 1 |
|  | b) Statistical test not appropriate, not described, or incomplete | 0 |

FACIT, Functional Assessment of Chronic Illness Therapy; ICD, International Classification of Diseases; ICU, intensive care unit; MoCA, Montreal Cognitive Assessment; NA, not applicable; NOS, Newcastle-Ottawa scale.

# References

1 Symons, M. J. & Moore, D. T. Hazard rate ratio and prospective epidemiological studies. *J Clin Epidemiol.* **55**, 893-899 (2002).

2 Wang, Z. Converting odds ratio to relative risk in cohort studies with partial data information. *J Stat Softw.* **55**, 1-11 (2013).

3 Hammel, I. S., Tosi, D. M., Tang, F., Pott, H. & Ruiz, J. G. Frailty as a risk factor for post-acute sequelae of COVID-19 among US veterans during the Delta and Omicron waves. *J Am Geriatr Soc.* **71**, 3826-3835 (2023).

4 Lundberg-Morris, L. et al. Covid-19 vaccine effectiveness against post-covid-19 condition among 589 722 individuals in Sweden: population based cohort study. *BMJ.* **383**, e076990 (2023).

5 Wander, P. L. et al. Rates of ICD-10 Code U09.9 documentation and clinical characteristics of VA patients with post-COVID-19 condition. *JAMA Netw Open.* **6**, e2346783 (2023).

6 Wu, Q. et al. Real-world effectiveness and causal mediation study of BNT162b2 on long COVID risks in children and adolescents. *EClinicalMedicine.* **79**, 102962 (2024).

7 Di Fusco, M. et al. Impact of COVID-19 and effects of booster vaccination with BNT162b2 on six-month long COVID symptoms, quality of life, work productivity and activity impairment during Omicron. *J Patient Rep Outcomes.* **7**, 77 (2023).

8 Razzaghi, H. et al. Vaccine effectiveness against long COVID in children. *Pediatrics.* **153**, e2023064446 (2024).

9 Li, J., Nadua, K., Chong, C. Y. & Yung, C. F. Long COVID prevalence, risk factors and impact of vaccination in the paediatric population: a survey study in Singapore. *Ann Acad Med Singap.* **52**, 522-532 (2023).

10 Mazzitelli, M. et al. Risk of hospitalization and sequelae in patients with COVID-19 treated with 3-day early remdesivir vs. controls in the vaccine and Omicron era: a real-life cohort study. *J Med Virol.* **95**, e28660 (2023).

11 Ballouz, T. et al. Post COVID-19 condition after Wildtype, Delta, and Omicron SARS-CoV-2 infection and prior vaccination: pooled analysis of two population-based cohorts. *PLoS One.* **18**, e0281429 (2023).

12 de Bruijn, S. et al. Lower prevalence of post-Covid-19 condition following Omicron SARS-CoV-2 infection. *Heliyon.* **10**, e28941 (2024).

13 Cortellini, A. et al. SARS-CoV-2 omicron (B.1.1.529)-related COVID-19 sequelae in vaccinated and unvaccinated patients with cancer: results from the OnCovid registry. *Lancet Oncol.* **24**, 335-346 (2023).

14 Di Fusco, M. et al. Effectiveness of BNT162b2 BA.4/5 bivalent COVID-19 vaccine against long COVID symptoms: a US nationwide study. *Vaccines (Basel).* **12**, 183 (2024).

15 Domenech-Montoliu, S. et al. Long COVID prevalence and the impact of the third SARS-CoV-2 vaccine dose: a cross-sectional analysis from the third follow-ip of the Borriana cohort, Valencia, Spain (2020-2022). *Vaccines (Basel).* **11**, 1590 (2023).

16 Hedberg, P. & Naucler, P. Post-COVID-19 condition after SARS-CoV-2 infections during the omicron surge vs the delta, alpha, and wild type periods in Stockholm, Sweden. *J Infect Dis.* **229**, 133-136 (2024).

17 Kahlert, C. R. et al. Post-acute sequelae after severe acute respiratory syndrome coronavirus 2 infection by viral variant and vaccination status: a multicenter cross-sectional study. *Clin Infect Dis.* **77**, 194-202 (2023).

18 Nehme, M. et al. Prevalence of post-coronavirus disease condition 12 weeks after omicron infection compared with negative controls and association with vaccination status. *Clin Infect Dis.* **76**, 1567-1575 (2023).

19 Reme, B. A., Gjesvik, J. & Magnusson, K. Predictors of the post-COVID condition following mild SARS-CoV-2 infection. *Nat Commun.* **14**, 5839 (2023).

20 Sun, X. et al. Postacute sequelae SARS-CoV-2 infection by vaccination status: a six-month latent class analysis. *medRxiv.* (2023).

21 Thaweethai, T. et al. Development of a definition of postacute sequelae of SARS-CoV-2 infection. *JAMA.* **329**, 1934-1946 (2023).

22 Herting, A. et al. Clinical outcomes of SARS-CoV-2 breakthrough infections in liver transplant recipients during the omicron wave. *Viruses.* **15**, 297 (2023).

23 Huh, K. et al. Vaccination and the risk of post-acute sequelae after COVID-19 in the Omicron-predominant period. *Clin Microbiol Infect.* **30**, 666-673 (2024).

24 Spiliopoulos, L. et al. Post-acute symptoms 4 months after SARS-CoV-2 infection during the Omicron period: a nationwide Danish questionnaire study. *Am J Epidemiol.* **193**, 1106-1114 (2023).

25 Richard, S. A. et al. Decreased self-reported physical fitness following SARS-CoV-2 infection and the impact of vaccine boosters in a cohort study. *Open Forum Infect Dis.* **10**, ofad579 (2023).

26 AlBahrani, S. et al. Self-reported long COVID-19 symptoms are rare among vaccinated healthcare workers. *J Infect Public Health.* **16**, 1276-1280 (2023).

27 Gallant, M., Mercier, K., Rioux-Perreault, C., Lemaire-Paquette, S. & Piché, A. Prevalence of persistent symptoms at least 1 month after SARS-CoV-2 Omicron infection in adults. *J Assoc Med Microbiol Infect Dis Can.* **8**, 57-63 (2023).

28 Zhou, S. & Shen, C. Avoiding definitive conclusions in meta-analysis of heterogeneous studies with small sample sizes. *JAMA Otolaryngol Head Neck Surg.* **148**, 1003-1004 (2022).

29 Brown, M. et al. Ongoing symptoms and functional impairment 12 weeks after testing positive for SARS-CoV-2 or influenza in Australia: an observational cohort study. *BMJ Public Health.* **1**, e000060 (2023).

30 Woldegiorgis, M., Cadby, G., Ngeh, S. & Korda, R. Long COVID in a highly vaccinated population infected during a SARS-CoV-2 Omicron wave – Australia, 2022. *medRxiv.* (2023).

31 Mikolajczyk R, D. S., Klee B, Pfrommer L, Purschke O, Fricke J, et al. Risk of post-COVID condition under hybrid immunity - results from the German National Cohort (NAKO). *SSRN.* **89**, 106206 (2023).

32 Congdon, S. et al. Nirmatrelvir/ritonavir and risk of long COVID symptoms: a retrospective cohort study. *Sci Rep.* **13**, 19688 (2023).

33 Antonelli, M. et al. SARS-CoV-2 infection following booster vaccination: illness and symptom profile in a prospective, observational community-based case-control study. *J Infect.* **87**, 506-515 (2023).

34 Diexer, S. et al. Association between virus variants, vaccination, previous infections, and post-COVID-19 risk. *Int J Infect Dis.* **136**, 14-21 (2023).
